# Circadian rhythms and lung cancer biology and immunotherapy: Emerging opportunities and challenges

**DOI:** 10.1016/j.pccm.2026.04.001

**Published:** 2026-06-09

**Authors:** Fang Tian, Songkai Wang, Zhe Huang, Zhaohui Ruan, Jiacheng Dai, Huan Yan, Jiao Huang, Dan Yang, Xiaomei Li, Liang Zeng, Qinqin Xu, Yongchang Zhang

**Affiliations:** a Early Clinical Trial Center/Office of National Drug Clinical Trial Institution, Hunan Cancer Hospital, The Affiliated Cancer Hospital of Xiangya School of Medicine, Central South University, Changsha, Hunan, 410013, China; b College of Clinical Medicine, Qinghai University, Xining, Qinghai, 810000, China; c Department of Pathology, School of Basic Medical Science, Central South University, Changsha, Hunan, 410013, China; d Third Xiangya Hospital, Central South University, Changsha, Hunan, 410000, China; e UPR "Chronotherapy, Cancers and Transplantation", Paris-Saclay University, Faculty of Medicine Kremlin Bicêtre, Le Kremlin Bicêtre, Val-de-Marne, 94270, France; f Department of Medical Oncology, Qinghai Provincial People's Hospital, Xining, Qinghai, 810000, China

**Keywords:** Circadian rhythm, Clock genes, Lung cancer, Tumor immune microenvironment, Immunotherapy, Immune checkpoint inhibitors, Tumorigenesis

## Abstract

The circadian rhythm is an intrinsic timekeeping system that precisely regulates a wide range of physiological and pathological processes. Its core molecular mechanisms and regulatory networks are gaining increasing attention in the field of lung cancer research. Substantial evidence indicates that circadian clock genes are not only deeply involved in lung cancer initiation and progression but also profoundly influence the dynamic remodeling of the tumor immune microenvironment (TIME) in lung cancer. This review summarizes the expression patterns and functional mechanisms of circadian clock genes in various cancers. It specifically elaborates on their regulatory roles in immune cell function, malignant behaviors of tumor cells, and interactions with stromal cells. The review also details how circadian disruption drives lung cancer development, progression, and immune evasion. Besides, we explore the potential impact of heterogeneity in circadian gene expression on the efficacy of immune checkpoint inhibitors (ICIs) in lung cancer, synthesize current knowledge on how circadian rhythms influence clinical responses to and adverse effects of immunotherapy in lung cancer, and highlight the translational potential of chronotherapy—optimizing drug administration timing based on circadian principles—to enhance therapeutic outcomes and reduce toxicity. By integrating recent preclinical and clinical evidence, this review provides a theoretical foundation for circadian-based precision immunotherapy in lung cancer and outlines future directions for innovative therapeutic strategies targeting the circadian machinery.

## Introduction

The circadian rhythm serves as a core mechanism enabling organisms to adapt to periodic environmental changes, regulating diverse biological processes including sleep, metabolism, and immunity.^1^, ^2^, ^3^ Its molecular foundation is built upon clock genes, which maintain 24-hour physiological rhythm through intricate transcription–translation feedback loops.^4^ This endogenous clock influences cell cycle progression, DNA repair, and inflammatory responses, and profoundly regulates immune system function. Research indicates that the integrity of the circadian rhythm is crucial for immune homeostasis: regular sleep and rhythmicity help optimize immune responses, whereas rhythm disruption can impair the body’s defense against infections and inflammation.^5^, ^6^, ^7^ Epidemiological studies have shown that long-term circadian rhythm disruption, such as that caused by shift work, jet lag, and irregular sleep patterns, can dysregulate cellular physiology and increase the risk of tumorigenesis.^7^^,^^8^ Among these factors, circadian disruption induced by shift work has been significantly associated with an increased incidence of lung cancer. Recent studies have further demonstrated a strong link between circadian disruption and the development of various diseases. During tumor initiation and progression, the circadian rhythm influences the biological properties of cancer cells by regulating multiple pathways, including cell proliferation, DNA damage repair, apoptosis, and metabolic reprogramming. In lung cancer tissues, the expression of core clock genes, such as circadian locomotor output cycles protein kaput (*CLOCK*), brain and muscle arnt-like 1 (*BMAL1*), period (*PER*), and cryptochrome (*CRY*), is significantly altered, and these abnormalities are closely associated with clinicopathological characteristics and patient prognosis. Functional studies have confirmed that clock genes regulate the proliferation, migration, and invasion of lung cancer cells, involving key pathways related to the cell cycle, apoptosis, and epithelial–mesenchymal transition. Beyond its direct effects on cancer cells, the circadian rhythm plays a significant role in shaping the tumor immune microenvironment. At the molecular level, clock genes regulate the migration, differentiation, and functional states of immune cells, thereby influencing immune cell infiltration and anti-tumor activity within the tumor microenvironment (TME). Furthermore, the circadian rhythm is closely linked to energy metabolism, regulating pathways related to glucose, lipids, and other metabolites, which subsequently impact the functional performance of immune cells in the TME.^6^^,^^7^^,^^9^

Cancer immunotherapy, particularly immune checkpoint inhibitors (ICIs), has become a cornerstone treatment for various malignancies, and has emerged as a core therapeutic strategy for driver-negative non-small cell lung cancer (NSCLC) and small cell lung cancer (SCLC). However, significant individual variation exists in the clinical efficacy of ICIs, with treatment responses often varying considerably even among lung cancer patients with identical pathological subtypes. Emerging evidence suggests that the circadian rhythm may be a critical factor influencing both the effectiveness and adverse effects of immunotherapy. The circadian rhythm not only affects the activity of immune cells and the immune status of the TME but may also influence the timing and safety of immunotherapies by modulating drug metabolism and the intensity of immune responses.^10^, ^11^, ^12^ For instance, aligning ICI administration with the patient’s circadian phase has been shown to significantly improve overall survival (OS) and progression-free survival (PFS), highlighting a close interaction between circadian rhythms and immunotherapy outcomes.^10^^,^^11^ In the field of lung cancer, retrospective studies have demonstrated that among patients with NSCLC, those receiving their first immunotherapy infusion in the morning had a significantly longer median overall survival compared to those treated later in the day. Prospective phase III clinical trials have now begun to explore this association, suggesting that controlling infusion timing during the initial treatment cycles may have a profound impact on long-term survival outcomes. Moreover, alterations in the expression of circadian rhythm-related genes have been linked to the tumor immune microenvironment, immune cell infiltration, and response to immunotherapy, providing a novel biological basis for developing personalized treatment strategies.^13^, ^14^, ^15^ Preliminary translational research indicates that circadian rhythm-based optimization of immunotherapy dosing schedules may enhance response rates to ICIs in NSCLC patients, offering new insights for precision treatment in lung cancer.

## Molecular basis and mechanisms of circadian rhythms

**Fig. 1:**
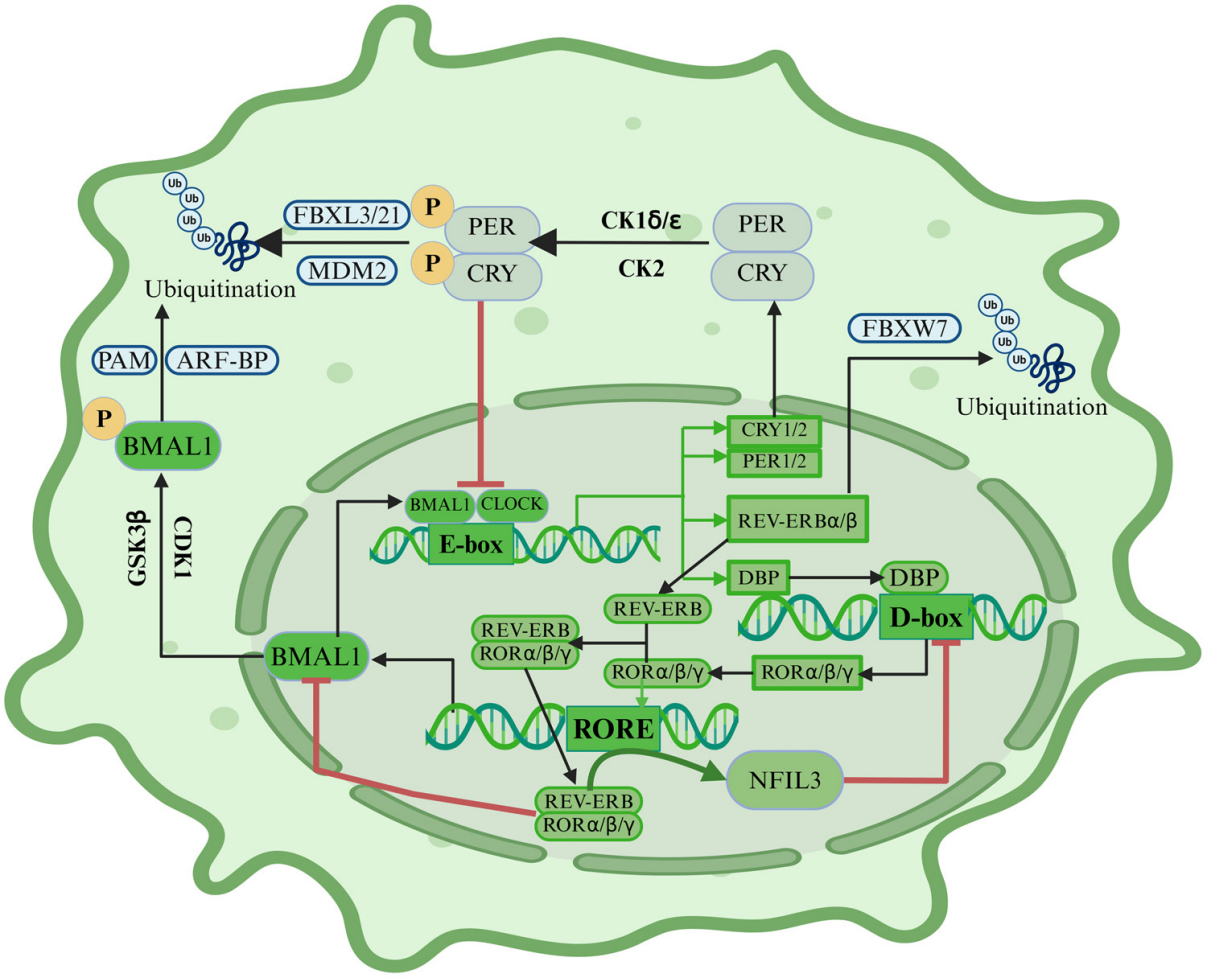
Molecular mechanism of transcription–translation feedback loops in the circadian clock. The core circadian oscillations are driven by feedback loops involving CLOCK, BMAL1, PER, and CRY. CLOCK and BMAL1 form a heterodimer complex that binds to E-box elements, activating the transcription of *PER, CRY, DBP*, and *REV-ERB*. Subsequently, PER and CRY proteins accumulate in the cytoplasm, where they are phosphorylated by CK1δ and CK1ε. The phosphorylated PER and CRY proteins then translocate into the nucleus and inhibit CLOCK–BMAL1-mediated transcription, thereby modulating the duration of cytoplasmic PER and CRY retention. Phosphorylated PER and CRY are targeted for degradation by E3 ubiquitin ligases, including FBXL3, FBXL21, and MDM2. In a secondary loop, DBP binds to d-box elements to regulate transcription of genes containing RORE elements. In a third regulatory circuit, RORα/β themselves activate BMAL1 transcription; however, upon forming transcriptional complexes with REV-ERB, they conversely suppress BMAL1 and promote NFIL3 expression. NFIL3, in turn, represses the transcription of RORα/β/γ. Following nuclear export of BMAL1, it undergoes phosphorylation by GSK3β and CDK1 in the cytoplasm. Both phosphorylated BMAL1 and REV-ERB are degraded via ubiquitination by E3 ligases such as ARF-BP, PAM, and FBXW7. ARF-BP, Also called HUWE1 (HECT, UBA and WWE domain containing E3 ubiquitin protein ligase 1); BMAL1, Brain and muscle ARNT-like 1; CDK1, Cyclin-dependent kinase 1; CK, Casein kinase; CLOCK, Circadian locomotor output cycles protein kaput; CRY, Cryptochrome; DBP, D-box binding protein; FBXL3/21, F-box and leucine-rich repeat protein 3/21; FBXW7, F-box and WD repeat domain containing 7; GSK3β, Glycogen synthase kinase-3 beta; MDM2, Mouse double minute 2; NFIL3, Nuclear factor interleukin 3; PAM, Also called MYCBP2 (MYC binding protein 2); PER, Period; REV-ERB, Reverse erythroblastosis virus; ROR, Retinoic acid-related orphan receptor; RORE, ROR response elemen; Ub, Ubiquitylation.

The internal biological clock in humans is composed of a sophisticated molecular network, with the suprachiasmatic nucleus (SCN) serving as the primary circadian pacemaker. The SCN exerts control over the organism through the rhythmic regulation of physiologic processes, including temperature, hormonal levels, and/or the autonomous nervous system.^16^ At its core, this intricate system comprises a complex transcription–translation feedback loop (TTFL) orchestrated by a set of core clock genes—including *CLOCK, BMAL1, PERs*, and *CRYs*—and their protein products. This molecular machinery collectively drives the 24-hour physiological rhythms of the organism, encompassing critical cyclical processes such as sleep–wake cycles and food intake.^17^ In mammals, CLOCK and BMAL1 proteins first form a heterodimer in the nucleus. This complex binds to E-box elements in the promoters of target genes, initiating the transcription of clock-controlled genes, including *PER* and *CRY*.^18^ Subsequently, PER and CRY proteins accumulate in the cytoplasm and form complexes; following phosphorylation, these complexes translocate into the nucleus and inhibit the transcriptional activity of the CLOCK/BMAL1 complex, thereby creating a negative feedback loop.^19^, ^20^, ^21^ The CRY/PER complex is eventually degraded via ubiquitination, relieving the inhibition on CLOCK/BMAL1. This cyclic process establishes an autoregulatory feedback loop that is central to circadian timing, ensuring the periodic expression of clock genes and their downstream targets, thereby maintaining 24-hour physiological rhythms (Fig. 1).^22^, ^23^, ^24^, ^25^

The clock gene regulatory network is not governed by a single loop but consists of multiple, interlocking positive and negative feedback circuits. For instance, the BMAL1/CLOCK heterodimer not only activates the transcription of *PER* and *CRY* but also induces the expression of auxiliary clock genes such as nuclear receptor subfamily 1 group D (*NR1D*) and d-box binding protein (*DBP*).^26^ These auxiliary genes, in turn, regulate *BMAL1* and *CLOCK* transcription through additional feedback pathways. For example, the nuclear receptors REV-ERBα and REV-ERBβ (encoded by *NR1D1* and *NR1D2*) can modulate DBP expression, which further promotes the transcription of retinoic acid receptor-related orphan receptors (RORs).^27^ RORα/β themselves can activate *BMAL1* transcription. However, when RORα/β form transcriptional complexes with REV-ERB, they can conversely suppress downstream *BMAL1* transcription and promote nuclear factor interleukin-3 (NFIL3) expression. NFIL3, in turn, can inhibit DBP-mediated activation of ROR transcription.^28^, ^29^, ^30^ Consequently, *BMAL1* expression is regulated by a complex feedback network involving REV-ERB and ROR. In this network, REV-ERB acts as a transcriptional repressor of *BMAL1* expression, whereas ROR positively regulates *BMAL1* expression by binding to the retinoic acid-related orphan receptor response element (RORE) in the *BMAL1* gene promoter. Furthermore, ROR itself is further modulated by the DBP/ROR–NFIL3/DBP loop as well as by REV–ERB. This multi-layered regulation enhances the precision and robustness of the circadian rhythm.^3^^,^^31^ Furthermore, the expression of clock genes is influenced by various internal and external signals, such as light, temperature, and metabolic status. Through interactions with key signaling pathway components like AMP-activated protein kinase (AMPK), mammalian target of rapamycin (mTOR), sirtuin 1 (SIRT1), and glycogen synthase kinase 3β (GSK3β), the circadian network can sense and adapt to environmental changes, enabling temporal regulation of downstream gene expression.^32^, ^33^, ^34^

## Causes of circadian disruption and associated health risks

Circadian disruption has become increasingly prevalent in modern society, primarily driven by factors such as shift work, insomnia, and environmental light pollution. Shift work, particularly night shifts and rotating schedules, often leads to a misalignment between an individual’s sleep–wake cycle and the natural light–dark cycle, causing widespread dysregulation of physiological systems. Faraut *et al*^35^ and Zhang *et al*^36^ investigated the profound physiological impacts of night-shift work on healthcare workers through two complementary dimensions: immuno-endocrine regulation and digital biomarkers, respectively. Zhang *et al*^36^ provided the first objective, real-world quantification of such effects using wearable sensors and computational modeling. Their findings indicated significantly reduced rest–activity rhythm indices and widespread obliteration of core body temperature circadian rhythms among night-shift workers. A high-risk subgroup—comprising 17.5% of the cohort—exhibited severe and persistent circadian disruption, a condition significantly associated with long-term night-shift exposure. This macro-level pattern of circadian misalignment was mechanistically elucidated in the micro-level immunological study by Faraut *et al*^35^ Their analyses revealed phase reversals in the circadian rhythms of key immune cells—such as lymphocytes and T-helper cells—and in the inflammatory cytokine IL-6 among night-shift nurses. These disruptions were specifically correlated with social jet lag and accumulated sleep debt. Consistent with these findings, accumulating epidemiological evidence indicates that shift workers are more prone to metabolic diseases such as obesity and diabetes, largely because work schedules and irregular eating patterns disturb the circadian system.^37^ Animal studies further corroborate that circadian misalignment can lead to aberrant secretion of gut hormones and aggravated insulin resistance, thereby impairing metabolic homeostasis.^38^ Furthermore, environmental light pollution, especially nighttime exposure to artificial light sources (e.g., urban lighting, electronic device screens), interferes with the endogenous biological clock by suppressing melatonin secretion from the pineal gland, thereby exacerbating circadian rhythm disturbance.^39^^,^^40^ In modern society, an increasing number of young people are experiencing circadian misalignment due to nighttime exposure to artificial light at night (ALAN).^41^ A recent global time-series study utilizing satellite-derived ALAN data (1992–2018) and Global Burden of Disease database systematically evaluated the association between ALAN exposure and lung cancer risk across 201 countries and regions worldwide.^41^ Using a distributed lag non-linear model, the study found a significant positive correlation between ALAN exposure and lung cancer incidence, with a distinct lag effect: after adjusting for socio-demographic index and smoking prevalence, the maximum relative risk occurred at an exposure intensity of 8.6 with a lag of 2.4 years (risk ratio [RR] = 1.05, 95% confidence interval [CI]: 1.02–1.07). Subgroup analysis revealed a stronger association in developed countries, with Oceania showing the highest effect (RR = 1.51). Potential mechanisms underlying ALAN exposure-induced lung cancer risk include: suppression of melatonin secretion (melatonin possesses antioxidant, immunomodulatory, and tumor-suppressive properties), direct disruption of clock gene expression (lung adenocarcinoma animal models demonstrate that clock gene mutations promote lung tumor growth), induction of sleep deprivation (a Finnish cohort study showed that sleep duration <7.5 hours is significantly associated with lung cancer risk), and impairment of immune surveillance function.^42^ This study provides important epidemiological evidence supporting ALAN exposure as a potential risk factor for lung cancer.^42^ A meta-analysis, including 10 cohorts, 7 case control studies, and over 170,000 individuals, highlighted an association between high exposure to ALAN and breast cancer risk, especially in premenopausal women and those with estrogen receptor-positive breast cancer.^43^ Moreover, a positive correlation was found between ALAN and thyroid cancer risk, with the association being stronger in women.^44^ Insomnia or chronic sleep deprivation is another significant cause of circadian disruption; it can induce short-term effects like elevated blood pressure and heightened stress responses, and is closely associated with an increased long-term risk of chronic conditions such as cardiovascular disease, obesity, and diabetes.^45^^,^^46^

At the physiological level, circadian disruption significantly impacts immunity, metabolism, and tumor susceptibility. Firstly, the circadian rhythm governs the functional rhythms of various immune cells (e.g., T cells, B cells, macrophages). Under conditions of disruption, immune defense capacity is diminished, and inflammatory responses are more readily activated, thereby increasing the risk of infections and chronic inflammatory diseases.^47^^,^^48^ Additionally, circadian disruption leads to impairments in glucose and lipid metabolism, manifesting as insulin resistance, dyslipidemia, and increased fat deposition, thereby promoting the development of metabolic syndrome.^49^^,^^50^ Regarding tumor susceptibility, circadian disruption can promote tumor initiation and progression by interfering with mechanisms such as cell cycle control, DNA repair, apoptosis, and immune surveillance. Both epidemiological and experimental studies have found that individuals experiencing long-term circadian disruption have a significantly elevated risk of developing cancer, accompanied by increased accumulation of immunosuppressive cells (e.g., myeloid-derived suppressor cells) in the tumor microenvironment, which exacerbates tumor immune evasion and drug resistance.^51^^,^^52^

## Mechanisms of core clock genes in lung cancer

### Regulation of lung cancer cell cycle, apoptosis, and autophagy by clock genes

**Fig. 2:**
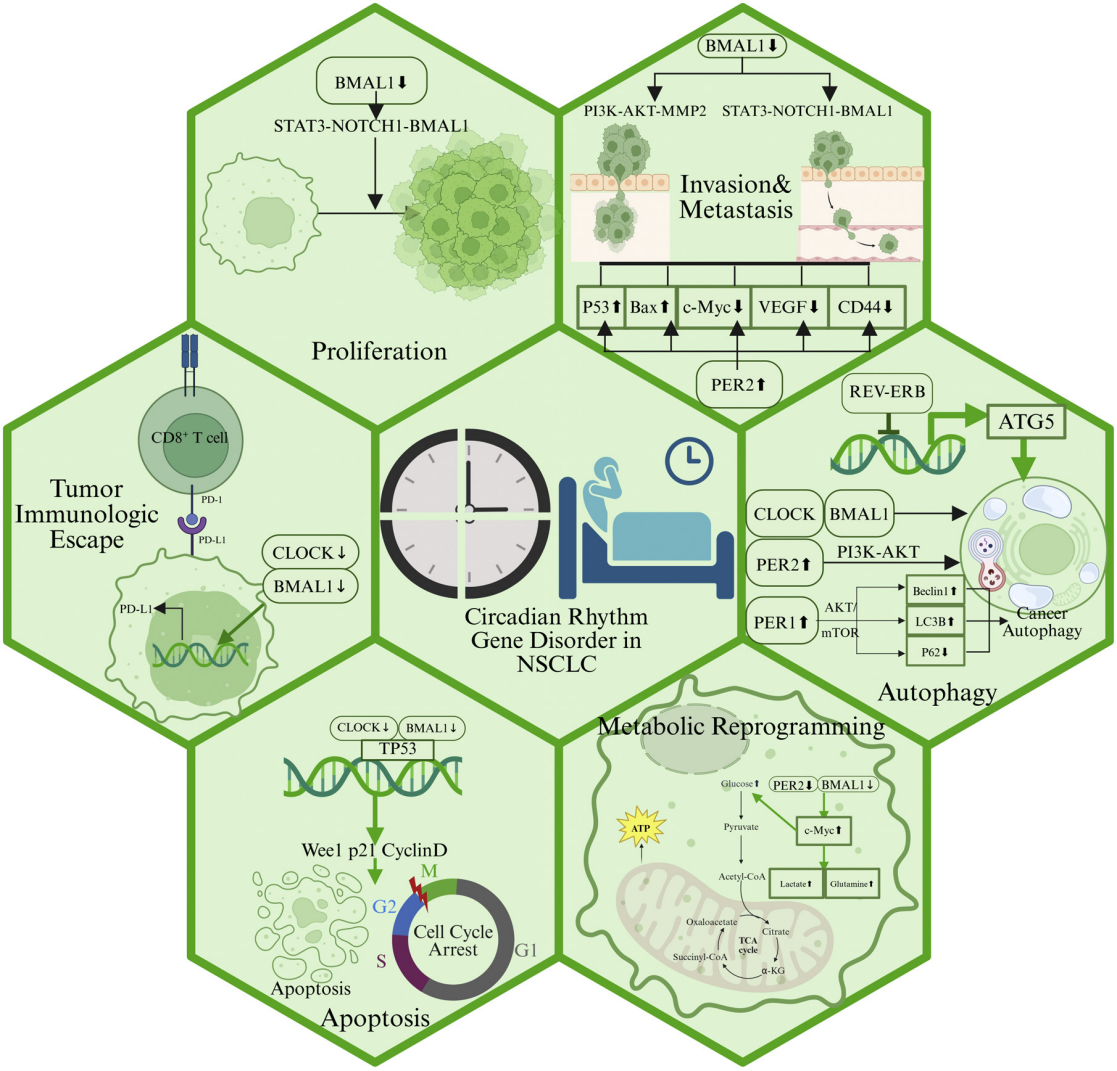
Effects of circadian rhythm disruption on lung cancer phenotypes. Disruption of the circadian rhythm promotes tumorigenesis, especially in lung cancer. Key malignant behaviors such as tumor cell proliferation, invasion, and metastasis are influenced by circadian dysregulation. Additionally, circadian clock genes including *CLOCK, BMAL1*, and *REV-ERB* participate in the regulation of immune escape and autophagy in tumors. Furthermore, processes such as apoptosis, cell cycle progression, and cellular metabolism are modulated by CLOCK and BMAL1. Loss of CLOCK and BMAL1 can induce apoptosis and cell cycle arrest via the p53 signaling pathway, while the metabolic reprogramming is frequently observed in tumor cells under circadian disruption. AKT, Serine/threonine kinase (protein kinase B, PKB); ATG5, Autophagy related 5; ATP, Adenosine triphosphate; Bax, BCL2 associated X protein; BMAL1, Brain and muscle ARNT-like 1; c-MYC, Cellular myelocytomatosis oncogene; CLOCK, Circadian locomotor output cycles kaput; LC3B, Light chain 3 beta; MMP2, Matrix metalloproteinase 2; mTOR, Mammalian target of rapamycin; NOTCH1, Notch receptor 1; NR1D, Nuclear receptor subfamily 1 group D member; NSCLC, Non-small cell lung cancer; PD-1, Programmed cell death protein 1; PD-L1, Programmed death ligand 1; PER1, Period circadian regulator 1; PER2, Period circadian regulator 2; PI3K, Phosphatidylinositol 3-kinase; REV, Reverse erythroblastosis virus; STAT3, Signal transducer and activator of transcription 3; TCA cycle, Tricarboxylic acid cycle; VEGF, Vascular endothelial growth factor.

Dysregulation of clock genes can lead to disrupted cell cycle rhythms, evasion of apoptosis, and imbalance in autophagy, collectively promoting tumorigenesis and development.^53^ For instance, tumor tissues often exhibit weakened or absent rhythmicity of clock genes, disrupted expression rhythms of cyclins, and dysregulation of apoptosis-related proteins (e.g., Caspase-3, Bcl-2 family) and oxidative stress responses, thereby enhancing tumor cell survival capacity (Fig. 2).^54^, ^55^, ^56^, ^57^

In regulating the cell cycle, the BMAL1/CLOCK heterodimer binds to the E-box element in the Wee1-like protein kinase (*WEE1*) gene promoter, directly activating its transcription and promoting WEE1 protein expression. Under conditions of DNA damage, WEE1 expression induces a protective G2/M phase arrest, thereby allowing time for DNA repair, inhibiting apoptosis, and enhancing cell survival.^58^ In the absence of DNA damage, however, BMAL1/CLOCK predominantly promotes cell cycle progression. In addition, BMAL1/CLOCK can indirectly suppress the expression of the cell cycle inhibitor p21 by activating transcriptional repressors such as REV-ERB, thereby preventing excessive cell cycle arrest that would otherwise impede normal proliferation. Thus, depending on the cellular context—whether DNA damage is present or not—BMAL1/CLOCK either transiently arrests the cell cycle via WEE1 to facilitate repair or drives proliferation through the coordinated downregulation of p21 and upregulation of Cyclin B1.^59^^,^^60^ Together, these dual mechanisms ensure the precise, context-dependent control of G2/M checkpoint dynamics under physiological conditions.

In contrast, loss of BMAL1/CLOCK also results in decreased expression of Cyclin B1 (CCNB1) and increased expression of the oncogene *Myc*, together exacerbating cell cycle dysregulation.^58^^,^^60^ Downregulation of REV-ERBα can promote cell proliferation and invasion via activation of the nuclear factor kappa-B (NF-κB) signaling pathway, leading to an increased proportion of G2-phase cells and an accelerated cell cycle.^61^^,^^62^ In *KRAS*-driven lung adenocarcinoma models, Papagiannakopoulos *et al*^63^ demonstrated that genetic disruption of *Per2* or *Bmal1* significantly promotes lung tumorigenesis, as evidenced by increased tumor burden and reduced survival, with tumor-derived cells showing accelerated proliferation and enhanced clonogenic potential. Mechanistically, *Per2* or *Bmal1* deficiency elevates cellular myelocytomatosis oncogene (c-Myc) expression and induces metabolic reprogramming characterized by increased glucose consumption, lactate production, and glutamine utilization with enhanced tricarboxylic acid cycle (TCA) cycle flux, thereby linking circadian disruption to uncontrolled proliferation.^63^ Moreover, core components of the cell cycle can reciprocally regulate the clock. In response to DNA damage or cellular stress, the tumor suppressor p53 is activated and directly binds to the p53 response element within the *PER2* promoter, which overlaps with the E-box, to suppress CLOCK/BMAL1-mediated transcription. This stress-induced inhibition of clock function represents a key mechanism by which p53 exerts its tumor-suppressive effects, overriding clock-driven proliferation to facilitate DNA repair or apoptosis when necessary. Notably, this regulatory interaction does not imply that CLOCK/BMAL1 itself is a tumor suppressor. Rather, it reflects the ability of p53 to act as an emergency brake that transiently overrides proliferative signals to maintain genomic integrity, regardless of whether those signals originate from clock-driven or other oncogenic pathways.^64^ Additionally, The G2/M phase master kinase cyclin-dependent kinase 1 (CDK1) can phosphorylate the clock protein REV-ERBα, facilitating its recognition and degradation by the F-box and WD repeat-domain containing 7 (FBXW7) ubiquitin ligase complex and consequently regulating the amplitude of circadian oscillations.^29^

In NSCLC, metabolic stress induced by glucose deprivation activates the 5′-adenosine monophosphate-activated protein kinase (AMPK) signaling pathway, leading to upregulation of BMAL1 expression. This upstream activation promotes the transcriptional upregulation of pro-apoptotic genes, including BCL2-associated X protein (Bax), initiator Caspase-8, and executioner Caspase-3, while simultaneously suppressing the expression of the anti-apoptotic protein Bcl-2. These coordinated molecular events activate the intrinsic mitochondrial apoptotic pathway and induce NSCLC cell apoptosis, thereby inhibiting tumor cell survival. In established NSCLC cell lines (*in vitro*) and formed subcutaneous xenograft tumors in nude mice (*in vivo*), activation of this pathway by glucose deprivation/ketogenic diet induces tumor cell apoptosis, thus indirectly suppressing tumor growth. This effect occurs during the treatment stage after tumor formation, rather than the tumor prevention stage.^65^ Pariollaud *et al*^66^ identified heat shock factor 1 (HSF1) as a key molecular link between circadian disruption and lung tumorigenesis. Their study demonstrated that chronic jet lag exposure disrupted the rhythmic expression of core clock genes including *Bmal1, Cry1*, and *NR1D1* in lung tissue, and led to dysregulated nuclear trafficking of HSF1, characterized by enhanced HSF1 accumulation at the beginning of the light phase and upregulation of its target genes, including the anti-apoptotic factor BCL2-associated athanogene 3 (Bag3). Notably, aberrant HSF1 activation accompanied by upregulation of its target genes may create a permissive microenvironment that facilitates early tumorigenesis. In esophageal squamous cell carcinoma (ESCC), PER2 expression exhibits circadian oscillation. During periods of low PER2 expression, apoptotic pathways are transcriptionally upregulated, enhancing cellular sensitivity to cisplatin through augmented DNA damage response, as evidenced by increased phospho-histone H2A.X (γH2AX) levels and apoptosis. This indicates that PER2 modulates apoptosis and cell cycle progression via regulation of the DNA damage response.^67^ Furthermore, the PER gene family interacts with the p53 pathway, cooperatively regulating the cellular response to DNA damage and apoptosis.^68^

In lung cancer, clock genes regulate autophagy through diverse and specific mechanisms to influence tumor progression. REV-ERB directly binds the autophagy related 5 (*ATG5*) promoter to inhibit its expression, thereby suppressing autophagy and delaying tumor progression.^69^ Shen *et al*^69^ further demonstrated that the REV-ERB agonist SR9009 exerts REV-ERB-dependent anti-lung cancer effects through autophagy inhibition, highlighting the critical role of REV-ERB in linking circadian regulation to autophagic control in lung cancer.

PER2 has also been implicated in autophagy regulation. Mechanistically PER2 can activate autophagy by recruiting tuberous sclerosis complex 1 (TSC1) to the mammalian target of rapamycin complex 1 (mTORC1) complex or enhance autophagic flux by suppressing the phosphatidylinositol 3-kinase (PI3K)/protein kinase B (AKT) pathway.^70^^,^^71^ Consistent with these pro-autophagic functions, knockdown of *Per2* suppresses autophagy in various cellular contexts.^72^^,^^73^ In addition, BMAL1 itself is subject to autophagic degradation, and this process is attenuated in the presence of a dominant-negative *CLOCK* mutant, suggesting that CLOCK may participate in autophagy regulation by facilitating autophagic turnover of BMAL1.^74^

In other tumor types, PER1 inhibits AKT/mTOR signaling, upregulates microtubule-associated protein 1 light chain 3B (LC3B) and Beclin1, downregulates P62, promotes autophagic flux, induces apoptosis and G2/M phase arrest; its loss leads to hyperactivation of AKT/mTOR, suppression of autophagy, and accelerated cell proliferation.^75^ These findings provide important insights for understanding clock gene-mediated autophagy regulation in lung cancer.

### Regulatory role of clock genes in tumor invasion and metastasis

Employing *KRAS*-driven murine models of lung adenocarcinoma, chronic circadian disruption was established by simulating shift work-induced jet lag. In KP mice (*Kras^G12D^; p53^flox/flox^*) exposed to jet lag, pulmonary tumor burden was substantially increased, accompanied by a higher proportion of high-grade tumors and a 9-day reduction in median survival.^63^ Parallel investigations in K mice (*Kras^G12D^*) confirmed that jet lag exposure elevated pulmonary tumor burden by 68%, predominantly characterized by increased tumor multiplicity rather than enlarged individual tumor size. These findings indicate that circadian rhythm disruption primarily affects early oncogenic events in *KRAS*-driven lung adenocarcinoma development.^2^

At the molecular mechanistic level, core circadian clock genes regulate the malignant phenotype of lung cancer cells through multiple pathways. BMAL1 suppresses matrix metalloproteinase-2 (MMP-2) expression in lung cancer cells by blocking the PI3K/AKT signaling pathway, thereby inhibiting cellular invasiveness. Jung *et al*^76^ validated in various lung cancer cell lines, including lung adenocarcinoma, that *BMAL1* knockdown activates the PI3K–AKT–MMP-2 pathway and promotes cellular invasion, whereas *BMAL1* overexpression significantly suppresses this process in a tumor protein p53 (p53)-independent manner. This contrasts with the role of BMAL1 in breast cancer, where it promotes invasion through MMP-9 upregulation.^77^ Studies by Xiang *et al*^78^ demonstrated that *PER2* overexpression in A549 lung adenocarcinoma cells not only significantly inhibits cell proliferation, migration, and invasion but also upregulates tumor suppressor genes including *Bax, TP53*, and cyclin-dependent kinase inhibitor 1A (*p21*), while simultaneously suppressing the expression of oncogenic factors such as vascular endothelial growth factor (VEGF), cluster of differentiation 44 (CD44), and c-Myc, thereby inhibiting lung cancer growth and metastasis both *in vitro* and *in vivo*. Furthermore, Jin *et al*^79^ found that l-theanine, an active component of green tea, suppresses lung adenocarcinoma cell stemness, cisplatin resistance, and migratory capacity by modulating the signal transducer and activator of transcription 3 (STAT3)/NOTCH1/BMAL1 signaling axis. Hepatic leukemia factor (HLF) exerts metastasis-suppressive functions in NSCLC through metabolic adaptation. Chen *et al*^80^ reported that HLF is significantly downregulated in early-recurrent NSCLC tissues, and its low expression is closely associated with early patient progression and distant metastasis. Mechanistic investigations revealed that HLF suppresses the NF-κB/p65 signaling pathway by promoting peroxisome proliferator-activated receptor α/γ (PPARα/PPARγ) nuclear translocation, thereby facilitating oxidative metabolism and reducing dependence on anaerobic glycolysis under nutrient-poor stress conditions, ultimately inhibiting lung cancer cell pulmonary colonization and multi-organ metastasis including bone, brain, and liver.^81^

Furthermore, circadian rhythm-related genes exert widespread functions in tumor proliferation and invasion across multiple malignancies. The PER family accelerates mitosis by regulating downstream cell cycle genes, thereby promoting tumor growth. BMAL1 enhances tumor cell invasiveness in breast cancer through upregulation of MMP-9,^77^ while high expression of CRY1 is associated with poor prognosis in colorectal cancer.^82^ These findings elucidate the complexity of the circadian rhythm regulatory network and its functional diversity across different tumor types, and provide important insights for deeper understanding of the tumor-specific regulatory mechanisms of circadian clock genes in lung cancer.

### Clock genes and tumor immune evasion in lung cancer

Dysregulation of circadian clock genes is closely associated with immune evasion in lung cancer. Pariollaud *et al*^66^ discovered in a *KRAS*-driven lung adenocarcinoma mouse model that chronic jet lag (CJL) exposure promotes lung cancer immune evasion through a dual mechanism: on one hand, CJL disrupts the circadian rhythm of neutrophil numbers, potentially weakening local immune surveillance by disrupting the temporal dynamics of immune cell migration; on the other hand, CJL selectively activates the HSF1 signaling pathway in lung adenocarcinoma epithelial cells, leading to accumulation of circadian clock disruptors in the nucleus, upregulation of anti-apoptotic factor Bag3, and the enrichment of HSF1-cancer signature (CaSig), thereby enhancing the anti-apoptotic capacity of tumor cells and facilitating their escape from immune attack.

At the molecular level, circadian clock factors directly regulate the expression of immune checkpoint molecules. Taking melanoma research as an example, the nuclear receptor RORA forms a complex with histone deacetylase 3 (HDAC3) under normal circadian rhythm and binds to the programmed death ligand 1 (*PD-L1*) promoter region to suppress its transcription; circadian rhythm disruption reduces RORA expression, relieving this suppression, leading to PD-L1 upregulation and driving T cell exhaustion.^83^ This mechanism provides important insights for understanding similar immune checkpoint regulation in lung cancer. Furthermore, circadian rhythm disruption promotes the remodeling of the tumor microenvironment toward an immunosuppressive state through multiple pathways, including reduced immune cell infiltration, impaired CD8^+^ T cell function, and alterations in metabolic and inflammatory pathways.^84^ Notably, the programmed cell death protein 1 (PD-1)/PD-L1 axis itself exhibits circadian oscillations in tumor-associated immune cells, which has direct implications for the efficacy of ICIs and the optimization of treatment timing.^85^ At the systemic level, circadian rhythm disruption can interfere with the circadian secretion of immunomodulatory factors such as cortisol and melatonin, creating conditions for immune evasion.^86^ Collectively, these findings reveal a complex multilayered network by which circadian rhythm disruption influences lung cancer immune evasion through multiple mechanistic pathways.

## Circadian regulation of the tumor immune microenvironment: Implications for lung cancer

**Fig. 3:**
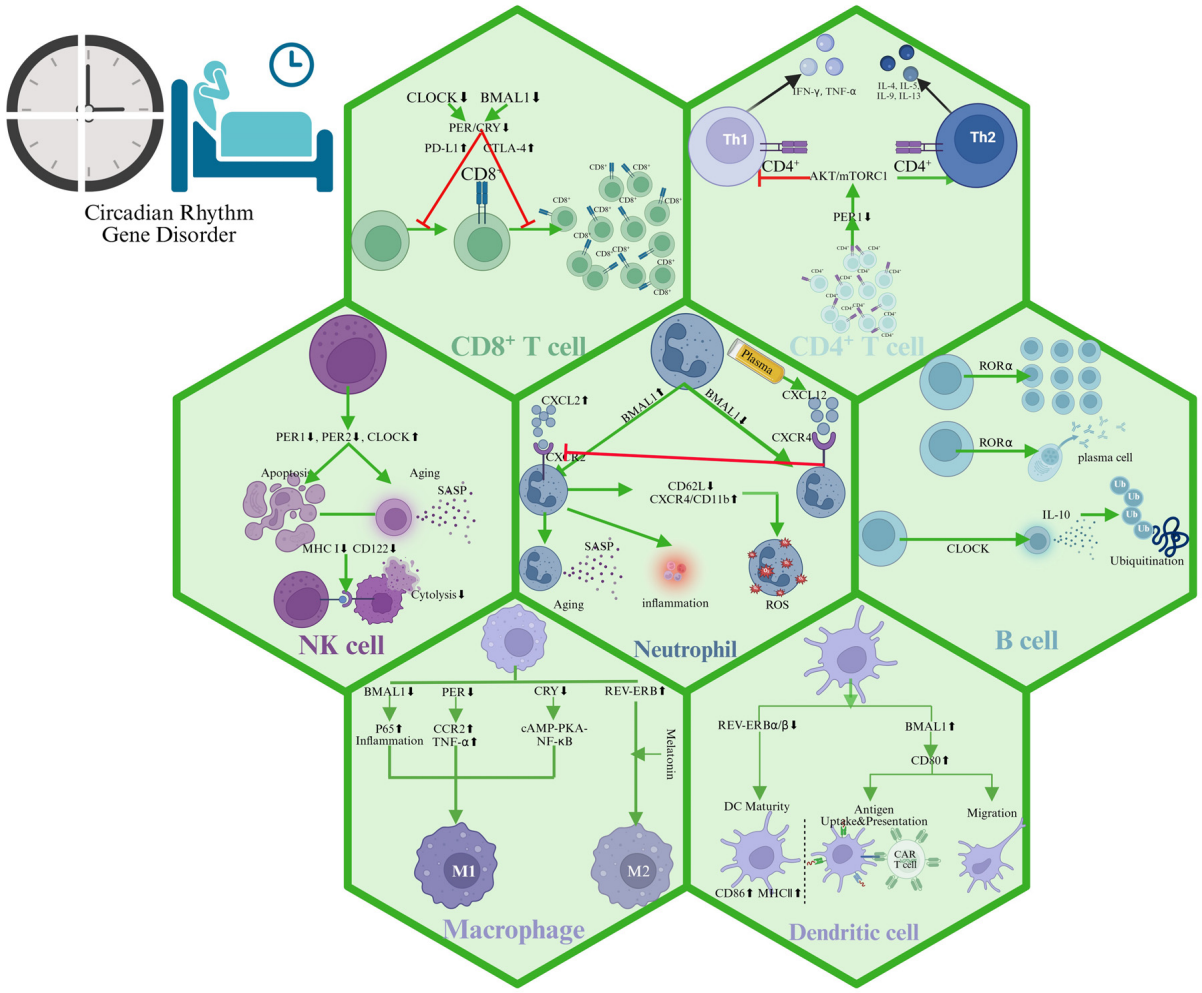
Circadian regulation of immune cell function. The function and responses of immune cells are regulated by core circadian clock components. In T cells, the PER/CRY complex modulates checkpoint molecules PD-L1/CTLA-4 in CD8⁺ T cells, while PER1 influences CD4⁺ T cell differentiation via the AKT-mTORC1 pathway. In NK cells, clock genes affect survival and cytotoxicity, partly through regulating MHC II and CD122 expression. In B cells, CLOCK interacts with c-Maf to promote IL-10 ubiquitination. In macrophages, genes including *BMAL1, REV-ERB, PER*, and *CRY* guide polarization and inflammatory outcomes. In dendritic cells, BMAL1 oversees antigen uptake, presentation, and migration. In neutrophils, the BMAL1-regulated CXCR4-CXCL12 axis modulates key functions including senescence, inflammation, and ROS production—a rhythm disrupted in the tumor microenvironment to promote cancer progression. AKT, Serine/threonine kinase (protein kinase B, PKB); BMAL1, Brain and muscle ARNT-like 1; cAMP, Cyclic adenosine monophosphate; CCR2, C-C chemokine receptor type 2; CD, Cluster of differentiation; CD11b, CD11 antigen-like family member B; CD62L, L-selectin; CLOCK, Circadian locomotor output cycles kaput; CRY, Cryptochrome; CAR-T, Chimeric antigen receptor T-cell; CTLA-4, Cytotoxic T lymphocyte-associated protein 4; CXCR4, C-X-C motif chemokine receptor 4; CXCL12, C-X-C motif chemokine ligand 12; IL, Interleukin; IFN-γ, Interferon γ; NK cell, Natural killer cell; MHC, Major histocompatibility complex; mTORC1, Mammalian target of rapamycin complex 1; NF-κB, Nuclear factor kappa-light-chain-enhancer of activated B cells; PD-L1, Programmed cell death ligand 1; PER, Period; PKA, Protein kinase A; RORα, Retinoic acid-related orphan receptor alpha; ROS, Reactive oxygen species; SASP, Senescence-associated secretory phenotype; Th1, T helper 1 cell; Th2, T helper 2 cell; TNF-α, Tumor necrosis factor α.

The circadian regulation of the TME has garnered increasing attention, as its homeostatic maintenance has been shown to profoundly dictate the magnitude and precision of anti-tumor immune responses. However, current research in this field remains largely concentrated on other malignancies, with relatively limited investigation into lung cancer to date. Given the pronounced immune heterogeneity and distinct therapeutic response patterns characteristic of lung cancer, deciphering how circadian rhythms orchestrate the tumor immune microenvironment is of paramount importance for optimizing immunotherapeutic strategies. Consequently, this section establishes a theoretical framework and provides strategic recommendations for investigating circadian dynamics within the lung cancer immune microenvironment (Fig. 3).^87^

### Circadian regulation of immune effector cells in lung cancer

#### T lymphocytes

As the central component of the adaptive immune system, T cell activation, differentiation, and cytokine secretion are precisely regulated by circadian rhythms. Approximately 6% of the CD8⁺ T cell transcriptome exhibits circadian oscillations, and both their abundance in the blood and their distribution in secondary lymphoid organs display robust 24-hour rhythmicity. This regulation stems from two sources: the intrinsic circadian clock mechanism within T cells themselves and the systemic modulation by rhythmic signals (such as hormones like cortisol).^88^

At the molecular level, T cells express core clock genes including *BMAL1, CLOCK*, and *PER1*. These genes maintain cell-autonomous circadian oscillations and directly regulate T cell function. For example, BMAL1 is essential for CD8⁺ T cell maturation, while deletion of CLOCK significantly impairs their proliferative capacity.^89^^,^^90^ In CD4⁺ T cells, PER1 suppresses the expression of T helper 1 (Th1)-type cytokines by inhibiting the AKT/mTORC1 signaling pathway and promotes differentiation toward the T helper 2 (Th2) and T helper 17 (Th17) lineages, thereby regulating the polarization of immune responses.^75^^,^^91^^,^^92^ Furthermore, clock genes regulate key molecules within the T cell receptor (TCR) signaling pathway (such as zeta-chain associated protein kinase 70 [ZAP70]), leading to time-dependent differences in antigen responsiveness.^93^ Homing receptors with rhythmic expression (e.g., interleukin 7 receptor [IL-7R] and C-X-C motif chemokine receptor 4 [CXCR4]) further modulate T cell retention and migration within lymphoid tissues.^94^^,^^95^

T cell responsiveness is highly time-dependent. In humans, naïve, effector, and memory CD4⁺ T cells, as well as naïve CD8⁺ T cells, peak in number at night and decline during the day, whereas effector CD8⁺ T cells show the opposite rhythm, peaking during the daytime.^96^ In nocturnal rodents, peripheral blood T cell counts peak during the resting phase (daytime, e.g., Zeitgeber time 4-5 [ZT4-5]) and reach their lowest levels during the active phase (nighttime, Zeitgeber time 16 [ZT16]).^88^ A key driver of this rhythmic migration is the circadian expression of the homing receptor C—C chemokine receptor 7 (CCR7) on T cells: Its nocturnal peak synchronizes with the rhythmic expression of its ligand C—C motif chemokine ligand 21 (CCL21) on high endothelial venules in lymph nodes, thereby driving the nocturnal accumulation of CD4⁺ and CD8⁺ T cells in lymph nodes.^97^, ^98^, ^99^ This rhythm persists under constant darkness conditions and can be abolished by T cell-specific knockout of *BMAL1*, confirming its endogenous circadian nature and cell-autonomous regulation.^97^

In lung cancer, the circadian characteristics of T cells are closely correlated with clinical treatment outcomes. Clinical studies have demonstrated that lung cancer patients receiving early-day immunotherapy exhibit superior treatment responses, characterized by a higher abundance of circulating CD8^+^ T cells and an elevated ratio of activated to exhausted CD8^+^ T cells. This suggests that optimizing the timing of treatment based on circadian rhythms can improve the prognosis of lung cancer patients by enhancing T cell function.^100^ Studies in other tumor types provide an important reference for understanding T cell regulation in lung cancer. For instance, in colon cancer models, the inhibition of the circadian clock protein RORα activates the NF-κB pathway and disrupts cholesterol homeostasis, which subsequently suppresses the activation and proliferation of CD8^+^ T cells and attenuates anti-tumor immunity.^101^ These findings indicate that similar regulatory mechanisms may also be present in lung cancer, warranting further experimental validation.

#### Natural killer (NK) cells

As a critical component of the innate immune system, NK cells exhibit pronounced circadian oscillations in both cytotoxic activity and cytokine secretion, with functional peaks occurring during the organism’s active phase—nocturnal in animal models such as mice and diurnal in humans. This rhythmic regulation is orchestrated by transcription-translation feedback loops driven by core clock genes, including *PER1, PER2*, and *CLOCK*, and plays an essential role in antitumor immunity.^102^^,^^103^ Human studies have further confirmed the circadian dependence of NK cell function: Systems immunology analyses reveal that the number and activity of peripheral blood NK cells display robust diurnal rhythms, with peak activity occurring in the afternoon, synchronized with the alertness of overall immune defense^104^; clinical and epidemiological evidence indicates that circadian disruption, such as night-shift work or sleep deprivation, reduces NK cell activity, thereby increasing the risk of infection and tumorigenesis.^105^ At the mechanistic level, circadian misalignment may also regulate NK cell function through epigenetic modifications, including m6A RNA methylation, potentially constituting a key downstream pathway through which clock genes exert sustained regulatory effects and further aggravate immune imbalance.^105^

In the field of lung cancer research, the regulatory role of circadian rhythms in NK cell function is receiving increasing attention. Logan *et al*^106^ demonstrated in a rat model that chronic shift-lag suppresses the rhythmic expression of NK cell effector molecules, including perforin and granzyme B, and promotes the pulmonary colonization and growth of MADB106 tumor cells, which are NK cell-sensitive and specifically metastasize to the lungs. Zeng *et al*^102^ further revealed in a mouse model that chronic shift-work downregulates Per1 and Per2 expression in NK cells, suppresses the transcription factor Eomes, and subsequently reduces transcription of the IL-15 receptor beta chain (CD122), thereby impairing NK cell responsiveness to IL-15 signaling. This leads to impaired NK cell development and accelerated senescence, characterized by an increased proportion of the CD27⁻CD11b⁺ senescent subset and reduced expression of activating Ly49 receptors, ultimately resulting in significantly impaired cytotoxicity and IFN-γ secretion, accompanied by enhanced B16 melanoma lung metastasis.^100^ These findings suggest that circadian rhythms may participate in pulmonary tumor immunosurveillance through the regulation of NK cell function. In other tumor types, clock gene expression has also been implicated in immune regulation: For example, in renal cell carcinoma (RCC) models, abnormal clock gene expression is not only associated with NK cell activity but may also contribute to remodeling of the tumor immune microenvironment, thereby influencing therapeutic responses.^103^ However, the specific role and clinical significance of the circadian rhythm–NK cell functional axis in lung cancer immunosurveillance remain poorly understood and warrant further investigation.

#### Neutrophils

Neutrophils possess an intrinsic, cell-autonomous circadian regulatory system. Under physiological conditions, the core of neutrophil circadian regulation involves the Bmal1-driven “aged/fresh” phenotypic switch. The transcription factor Bmal1 directly upregulates the chemokine C-X-C motif chemokine ligand 2 (CXCL2), which activates its receptor C-X-C motif chemokine receptor 2 (CXCR2), driving neutrophils to adopt an “aged” phenotype during the active phase (e.g., mouse ZT5), characterized by downregulated l-selectin (CD62L) and upregulated C-X-C motif chemokine receptor 4 (CXCR4) and CD11b. This phenotype is accompanied by a reduction in surface microvilli, impairing their ability to effectively roll and adhere to inflamed endothelium via selectins. Consequently, they migrate into homeostatic tissues via non-rolling mechanisms to perform clearance and protective functions.^107^^,^^108^ The key circadian switch is mediated by the CXCR4–CXCL12 axis. During the rest phase (e.g., mouse ZT13), elevated plasma CXCL12 bind to CXCR4, promoting the homing and clearance of aged neutrophils, while the bone marrow releases new cells.^109^ This replenishes the circulation with a “fresh” phenotype with intact microvilli, enabling effective rolling and migration to sites of inflammation and significantly enhancing anti-infective capacity.^107^^,^^108^ This phenotypic switching demonstrates clear physiological significance: Mice with neutrophil-specific deletion of Bmal1 lose circadian differences in infection resistance, whereas mice with neutrophil-specific CXCR4 deficiency fail to clear aged cells, leading to a sustained “aged” state in circulation and heightened inflammatory damage.

Within the tumor microenvironment, this intricate physiological circadian network is subverted by both systemic disruption and functional hijacking of the CXCL12 signal, collectively driving malignant progression. Functional hijacking of the CXCL12 signal is evidenced by the frequently sustained high expression of CXCL12 by tumor cells and the surrounding microenvironment. This property likely hijacks the physiological function of the CXCR4–CXCL12 axis, abnormally recruiting and educating neutrophils to accumulate within the tumor core. These educated neutrophils foster an immunosuppressive microenvironment conducive to tumor growth by releasing immunosuppressive and pro-angiogenic factors, rather than performing their physiological protective roles.^108^ Systemic disruption is primarily mediated by chronic stress, which via the glucocorticoid–glucocorticoid receptor (GR) axis directly upregulates the expression of clock genes such as *Per1* and *Per2*. This leads to neutrophils exhibiting a persistent “aged” phenotype (sustained high CXCR4 and low CD62L expression) accompanied by dysregulated reactive oxygen species (ROS) levels. Ultimately, this disrupts the circadian constraint on neutrophil extracellular trap (NET) formation, shifting it to a state of sustained high release.^110^ These NETs deposit in distant organs (particularly the lungs), promoting the accumulation of fibronectin to form a pro-metastatic extracellular matrix while inhibiting the infiltration of cytotoxic T cells. Thereby, they create a critical pre-metastatic niche that directly drives the metastatic process.^110^

However, research on the aforementioned circadian regulatory mechanisms of neutrophils in lung cancer remains in its infancy. Although evidence indicates that neutrophils play a critical role in the formation of the pulmonary pre-metastatic niche, whether they participate in remodeling the lung cancer immune microenvironment through rhythmic “aged/fresh” phenotypic switching has not been systematically reported. Given that lung cancer patients frequently experience sleep disturbances and chronic stress, further investigation into the role of the circadian rhythm–neutrophil functional axis in lung cancer pathogenesis and therapeutic response may offer novel intervention targets for chronotherapy in lung cancer.

### Regulation of antigen-presenting cells by circadian rhythms in lung cancer

#### Dendritic cells

Dendritic cells (DCs), as pivotal bridges linking innate and adaptive immunity, exert antigen-presenting functions that are precisely regulated by circadian rhythms, a process dependent on intrinsic clock genes.^111^ Studies have demonstrated that mitochondrial morphology and metabolic activity in DCs exhibit circadian oscillations, with BMAL1 serving as a key mediator of this rhythmic metabolism.^112^ Beyond intracellular metabolic regulation of DC function, the migratory and communicative capacities of DCs are likewise subject to circadian control.

Wang *et al*^113^ inoculated melanoma cells into mice at six distinct time points across the day (morning ZT1, midday ZT5, afternoon ZT9, evening ZT13, midnight ZT17, and late night ZT21) and found that mice inoculated in the afternoon (ZT9–ZT13) developed the smallest tumors, whereas those inoculated at late night (ZT21) exhibited the largest tumor volumes. This phenomenon was consistently recapitulated across multiple tumor models, including breast cancer and colorectal cancer, confirming its universality across tumor types. To further exclude confounding effects of external light–dark cycles, the research team housed mice under constant darkness conditions, allowing free-running of the endogenous circadian clock. Under these conditions, mice inoculated during the subjective afternoon still developed significantly smaller tumors than those inoculated during the subjective late night, thereby confirming the endogenous circadian nature of this phenomenon, driven by the organism’s intrinsic biological clock rather than passive responses to external photoperiodic cues.

Further investigation revealed that this time-dependent differential is driven by the intrinsic circadian clock within DCs. DCs from REV-ERBα and REV-ERBβ deficient mice showed enhanced expression of maturation markers like CD86, MHCII, and proinflammatory cytokines. Mice inoculated in the afternoon exhibited increased DC infiltration at the tumor site, along with significantly elevated numbers of CD103^+^ DCs and antigen-specific DCs in the draining lymph nodes. DC-specific deletion of Bmal1 completely abrogated the circadian differences in T cell proliferation and tumor growth.^113^^,^^114^ At the molecular level, BMAL1 directly binds to the promoter region of the co-stimulatory molecule CD80, driving its rhythmic expression and thereby modulating DC-mediated T cell activation capacity. Consistent with these mechanisms, afternoon tumor vaccine administration conferred significantly greater suppression of tumor growth compared with late-night inoculation. Notably, mice are nocturnal animals, and their “afternoon” corresponds to the rest phase, whereas humans, as diurnal animals, have a circadian rhythm that is opposite to that of mice. Consistent with this, retrospective analysis of melanoma patients further suggested that morning vaccine recipients exhibited higher levels of antigen-specific CD8⁺ T cells.^113^, ^114^, ^115^

Although the aforementioned findings have been established primarily in melanoma models, the central conclusion they reveal, namely that rhythmic DC function determines the temporal efficacy of antitumor immune responses, provides important conceptual insights for analogous investigations in the field of lung cancer. Nevertheless, whether the circadian rhythmicity of DC migration, antigen presentation, and T cell activation capacity similarly influences immune surveillance and therapeutic efficacy in lung cancer remains to be directly demonstrated. Future studies may draw upon experimental paradigms established in melanoma research, including multi-timepoint inoculation protocols and DC-specific circadian regulation strategies, to systematically investigate the impact of DC circadian rhythms on tumor progression and immunotherapeutic responses in lung cancer models, thereby providing experimental foundations for chronotherapeutic strategies in lung cancer management.

#### Macrophages

Macrophages, as key components of the innate immune system, exhibit pronounced circadian oscillations in cytokine secretion, phagocytosis, and antigen presentation. BMAL1 deficiency abolishes phagocytic rhythmicity and results in constitutively enhanced phagocytic activity, indicating that BMAL1 functions as a suppressive regulator of phagocytic response magnitude.^116^ Macrophage-mediated inflammatory responses display a circadian pattern characterized by stronger activity during the day and attenuated activity at night, a phenomenon closely associated with the rhythmic expression of circadian clock genes.^111^^,^^117^^,^^118^ Macrophage polarization is subject to circadian regulation. Under physiological conditions, M1 macrophages predominate during the daytime, whereas M2 macrophages are favored at night. Notably, compared with M1 macrophages, M2 macrophages exhibit a more robust circadian rhythm, characterized by higher amplitude and greater rhythm persistence, whereas M1 macrophages display a shortened circadian period.^119^^,^^120^

At the mechanistic level, core clock proteins such as BMAL1 and REV-ERBα function as direct repressors of M2 polarization.^121^^,^^122^ Consequently, circadian disruption—through the loss of these repressive signals—leads to aberrant skewing toward the M2 phenotype, which may promote the establishment of an immunosuppressive tumor microenvironment and drive tumor growth and metastasis.^123^^,^^124^

At the metabolic level, BMAL1 loss upregulates hypoxia-inducible factor 1 alpha (HIF-1α), thereby suppressing mitochondrial metabolism, promoting ROS accumulation, and inhibiting nuclear factor erythroid 2-related factor 2 (NRF2) signaling, ultimately altering pro-inflammatory cytokine production. In melanoma models, BMAL1-deficient macrophages exhibit impaired capacity to sustain mitochondrial oxidative phosphorylation, leading to elevated mitochondrial ROS, HIF-1α-driven glycolytic reprogramming, establishment of an immunosuppressive tumor microenvironment, and accelerated tumor progression.^123^ BMAL1 deficiency also augments lactate production, driving macrophage conversion toward a tumor-associated macrophage (TAM)-like phenotype.^123^^,^^124^ Notably, M1 macrophages display weaker circadian rhythmicity compared to M2 macrophages, and the acidic tumor microenvironment further modulates this characteristic: under pH 6.5 conditions, M2 rhythmicity is enhanced whereas M1 rhythmicity is attenuated.^125^^,^^126^ Approximately 8–15% of macrophage genes exhibit circadian rhythmic expression, and Toll-like receptor signaling is likewise subject to circadian regulation.^120^^,^^127^

Key circadian proteins regulate macrophage polarization in a context-dependent manner: At the level of basal immune regulation, BMAL1 and PER/CRY complexes suppress classical M1 polarization; REV-ERBα activation promotes M2 polarization and restrains inflammation via the PI3K/AKT pathway;^128^, ^129^, ^130^ CRY proteins negatively regulate the cyclic adenosine monophosphate (cAMP)/protein kinase A (PKA)/NF-κB axis, and their loss results in heightened inflammatory cytokine expression.^131^, ^132^, ^133^

#### B lymphocytes

As cornerstones of humoral immunity, B cells play an indispensable role in tumor immune responses and immunotherapy. Circadian rhythms profoundly influence B cell differentiation, antibody production, and memory B cell formation by regulating the expression of clock genes. Accumulating evidence indicates that circadian disruption not only alters B cell quantities but also reshapes their functional states: CLOCK upregulation restricts IL-10 transcription in regulatory B cells (B10 cells), impairing their immunomodulatory function and capacity to suppress T cell proliferation.^134^ Animal studies have further revealed that circadian misalignment induces significant alterations in splenic B cell subsets and upregulation of activation markers (e.g., CD69, major histocompatibility complex [MHC] II-related molecules).^135^ Given the importance of B cells in tumor immunity, we propose that circadian misalignment-induced B cell changes may influence anti-tumor immune responses.

The impact of circadian rhythms on B cell function within the TME is equally significant. Studies utilizing colorectal cancer models have demonstrated that the circadian clock in intestinal epithelial cells is crucial for maintaining local B cell homeostasis: Specific knockout of the core clock gene *BMAL1* remodels B cell subset composition within the TME, characterized by reduced naïve B cells and increased proportions of mature and proliferating B cells.^136^ Furthermore, circadian-related genes such as *RORA* have been implicated in B cell proliferation and differentiation: RORA deficiency leads to aberrant B cell accumulation in peripheral blood, bone marrow, and spleen, whereas its activation reduces B cell numbers, suggesting a suppressive role in B cell development.^137^

Although the aforementioned findings reveal the important regulatory role of circadian rhythms in B cells, these studies have primarily focused on autoimmune diseases and colorectal cancer models, leaving the specific role of circadian regulation of B cells in the lung cancer immune microenvironment largely unknown. Given the critical importance of tertiary lymphoid structures in lung cancer prognosis and response to immunotherapy, investigating how circadian B cell functions influence lung cancer progression and therapeutic efficacy may provide a novel scientific basis for optimizing lung cancer immunotherapy strategies.

## Impact of ICI administration timing on efficacy and adverse events in lung cancer

**Fig. 4:**
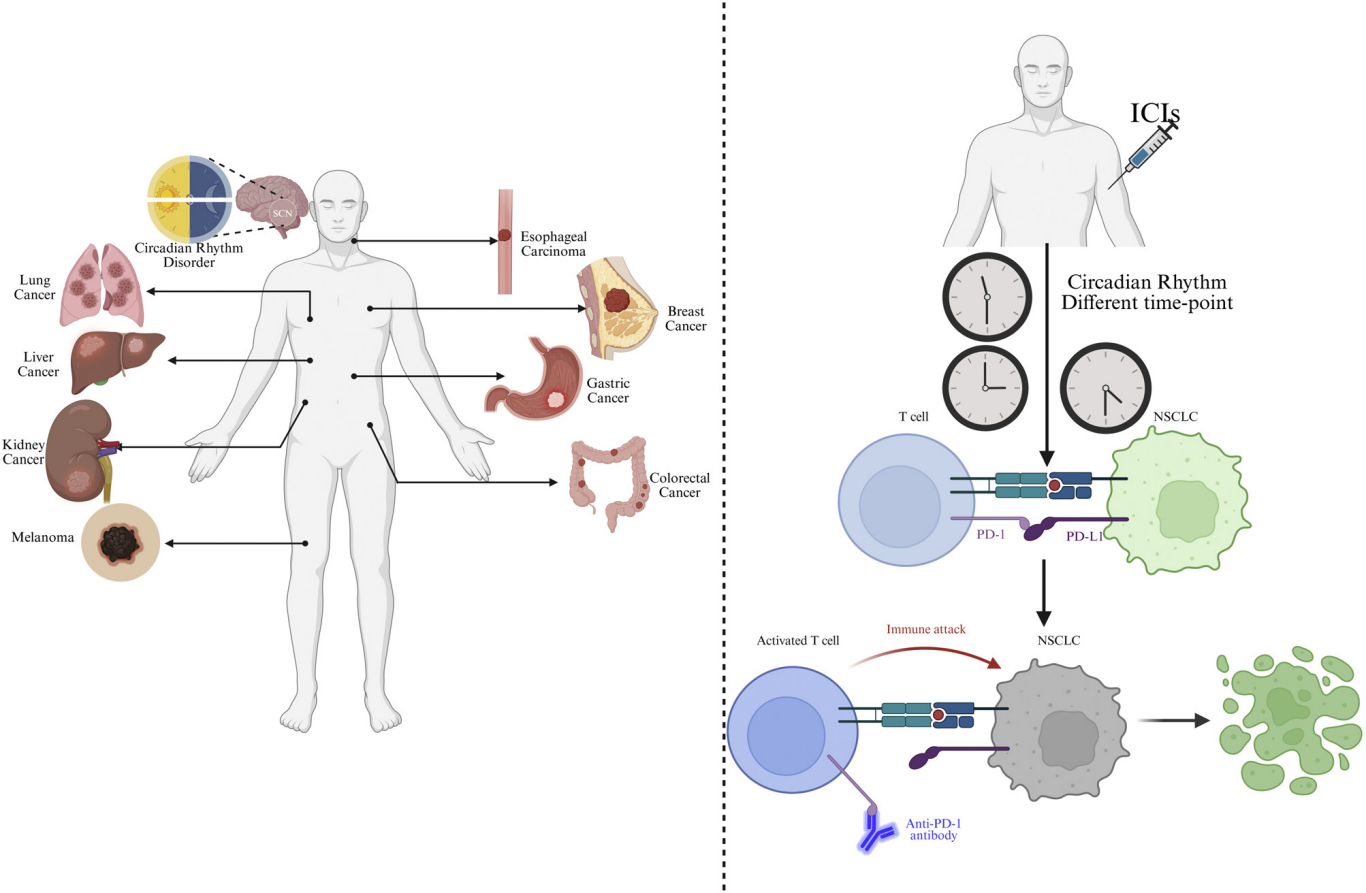
Circadian disruption as a driver of tumorigenesis in NSCLC and other malignancies. The mammalian central pacemaker, located in the SCN of the hypothalamus, processes photic input signals and transmits timing information to peripheral clocks. Circadian rhythm disruption has been implicated in the pathogenesis and progression of various cancers, which is largely associated with the dysregulation of core circadian clock molecules. In the left figure, this association between circadian rhythm and therapy has been reported in multiple malignancies, including lung, liver, kidney, and esophageal cancers, as well as melanoma, breast, gastric, and colorectal cancers. While in the right figure, the preliminary findings on the relationship between ICIs infusion timing and clinical outcomes in NSCLC have revealed a consistent pattern, whereas such an association appears less evident or remains insufficiently substantiated in other tumor types, suggesting that the efficacy of immunotherapy is time-dependent in a tumor type-specific manner. ICIs, Immune checkpoint inhibitors; NSCLC, Non-small cell lung cancer; PD-1, Programmed cell death 1; PD-L1, Programmed cell death ligand 1; SCN, Suprachiasmatic nucleus.

Tumor immunotherapy is among the most groundbreaking advancements in oncology in recent years. Distinct from traditional surgery, radiotherapy, and chemotherapy, immunotherapy works by activating the patient’s own immune system and enhancing its ability to recognize and eliminate tumor cells, achieving significant clinical efficacy across various malignant tumors. In the field of lung cancer, ICIs have emerged as the core therapeutic strategy for driver gene-negative NSCLC and SCLC, significantly improving patient survival outcomes (Fig. 4). Despite its transformative potential, immunotherapy still faces challenges such as heterogeneity in treatment response, immune resistance, and immune-related adverse events, which are particularly prominent in lung cancer patients.

Chronotherapeutics, a science that optimizes the timing of medical interventions based on biological rhythms, provides novel insights for improving and optimizing cancer treatment. This discipline is based on the understanding that physiological processes, including drug metabolism, cell proliferation, and immune system activity, exhibit predictable diurnal fluctuations.^138^ By synchronizing drug administration with these endogenous rhythms, chronotherapeutics aims to maximize efficacy while minimizing adverse reactions. In oncology, this concept has been explored with various traditional chemotherapeutic agents.^139^ With the widespread clinical application of ICIs, it has been increasingly recognized that although ICIs have significantly improved treatment outcomes for various malignancies, their efficacy remains constrained by substantial heterogeneity. Among NSCLC patients, treatment responses often vary significantly even within the same pathological type.

Recent studies have demonstrated that circadian rhythms, acting as an intrinsic “time coder,” profoundly influence the spatiotemporal distribution of ICI efficacy by regulating immune cell activity and remodeling the tumor microenvironment. This paradigm has been particularly well-validated in lung cancer. Importantly, the impact of circadian rhythms on ICI efficacy appears to be tumor type-dependent, and as a primary focus of circadian research, lung cancer possesses the most abundant relevant evidence. Multiple retrospective clinical studies have investigated the impact of ICI infusion timing on treatment outcomes in patients with NSCLC, melanoma, esophageal cancer, hepatocellular carcinoma, RCC, gastric cancer, and head and neck squamous cell carcinoma (Table 1). Therefore, determining the optimal administration timing for ICIs is crucial for maximizing their therapeutic benefits. Particularly in lung cancer, a core indication for immunotherapy, optimizing the timing of drug administration holds the promise of further enhancing the clinical value of immunotherapeutic strategies.

**Table 1:** Retrospective clinical studies on immunotherapy across various malignancies.

Study	Tumor type	ICI agent	TOD cut off	Grouping strategy	Number of patients (before/after)	Endpoint	Before *vs.* After (months, unless otherwise specified)
Guo X *et al*^140^	NSCLC	Tislelizumab Pembrolizumab Camrelizumab	12:00	Median infusion time	36/37	PFS, ORR	mPFS: 9.8 *vs.* 16.5, *P* = 0.031 ORR: 8.3% *vs.* 25.0%, *P* = 0.308
Karaboué A *et al*^141^	NSCLC	Nivolumab	12:54	Median infusion time	48/47	PFS, OS, ORR	mPFS: 11.3 *vs.* 3.1, *P* < 0.001 mOS: 34.2 *vs.* 9.6, *P* < 0.001 ORR: 37.5% *vs.* 14.4%, *P* = 0.031
Huang Z *et al*^143^	NSCLC	Pembrolizuma Sintilimab Toripalimab Tislelizumab Camrelizumab	11:30	Median infusion time	345/368	PFS, OS, ORR	mPFS: 11.8 *vs.* 7.2, *P* < 0.0001 mOS: 33.0 *vs.* 19.5, *P* < 0.0001 ORR: 61.7% *vs.* 52.4%, *P* = 0.002
Cortellini A *et al*^144^	NSCLC	Pembrolizumab	16:30	Before group: <20% after cutoff After group: ≥20% after cutoff	136/44	OS, PFS	mPFS: 19.7 *vs.* 6.6, *P* = 0.056 mOS: 47.1 *vs.* 27.8, *P* = 0.11
Rousseau A *et al*^145^	NSCLC	PD-1/PD-L1	16:30	Before group: <20% after cutoff After group: ≥20% after cutoff	115/65	OS, PFS	mPFS: 9.4 *vs.* 4.9, *P* = 0.02 mOS: 26.2 *vs.* 14.0, *P* = 0.09
Hirata T *et al*^146^	NSCLC	Durvalumab	15:00	Before group: <20% after cutoff After group: ≥20% after cutoff	70/12	OS, PFS	mPFS: NA *vs.* 7.4, *P* = 0.027 mOS: NA *vs.* 22.4, *P* = 0.20
Huang Z *et al*^142^	SCLC	Atezolizumab Durvalumab	15:00	Median infusion time	344/53	PFS, OS, ORR	mPFS: 7.6 *vs.* 5.8, *P* < 0.001 mOS: 18.4 *vs.* 11.6, *P* < 0.001 ORR: 85.8% *vs.* 67.9%, *P* = 0.011
Fletcher K *et al*^147^	Melanoma	Nivolumab Pembrolizumab Ipilimumab+PD-1	16:00	Before group: <20% after cutoff After group: ≥20% after cutoff	318/198	PFS, OS, ORR	mPFS: 38.9 *vs.* 10.6, *P* < 0.0001 mOS: 81.2 *vs.* 54.6, *P* = 0.19 ORR: 46.9% *vs.* 31.9%, *P* = 0.001
Qian DC *et al*^148^	Melanoma	Ipilimumab Nivolumab Pembrolizumab	16:30	Before group: <20% after cutoff After group: ≥20% after cutoff	73/73	OS, 1-year PFS rate, ORR	OS: NA *vs.* 4.8 years, *P* = 0.038 1-year PFS rate:56.0% *vs.* 40%, *P* = 0.041 ORR: 34.0% *vs.* 22.0%, *P* = 0.069
Amici M *et al*^149^	Melanoma	Ipilimumab Nivolumab Pembrolizumab	11:00	Before group: <50% after cutoff After group: ≥50% after cutoff	56/98	OS, PFS	mPFS: 26.5 *vs.* 22.3, *P* = 0.945 mOS: 70.8 *vs.* NA, *P* = 0.761
Yeung C *et al*^150^	Melanoma	Nivolumab Pembrolizumab Ipilimumab+PD-1	13:00	Before group: At least one infusion before the cutoff After group: All four infusions after the cutoff	98/23	PFS, OS, ORR	mPFS: 7.6 *vs.* 3.3, *P* = 0.009 mOS: 24.9 *vs.* 5.5, *P* < 0.001 ORR: 36.0% *vs.* 14.0%, *P* = 0.073
Ishizuka Y *et al*^151^	Metastatic gastric cancer	Nivolumab	14:00	Before group: <70% after cutoff After group: ≥70% after cutoff	140/108	PFS, OS, ORR	mPFS: NA, HR = 0.67, *P* = 0.020 mOS: NA, HR = 0.57, *P* = 0.033 ORR: 34.0% *vs.* 21%, *P* = 0.044
Tanaka T *et al*^152^	Metastatic gastric cancer	Nivolumab	11:41	Median infusion time	29/29	PFS, OS, ORR	mPFS: 2.6 *vs.* 1.6, *P* = 0.017 mOS: 8.2 *vs.* 5.3, *P* = 0.016 ORR: 17.0% *vs.* 3.0%, *P* = 0.01
Cheng C *et al*^153^	Metastatic gastric cancer	Sintilimab Nivolumab Pembrolizumab Tislelizumab	16:30	Before group: <20% after cutoff After group: ≥20% after cutoff	164/50	PFS, OS, ORR	mOS: 22.5 *vs.*15.4, *P* = 0.013 PFS: NA, *P* = 0.190 ORR: 41% *vs.* 36%, *P* = 0.653
Patel JS *et al*^154^	Renal cell carcinoma	Nivolumab Nivolumab + Ipilimumab Pembrolizumab	12:00	Before group: <20% after cutoff After group: ≥20% after cutoff	101/100	PFS, OS, ORR	PFS: NA, HR = 0.67, *P* = 0.020 OS: NA, HR = 0.57, *P* = 0.033 ORR: 34.0% *vs.* 21%, *P* = 0.044
Dizman N *et al*^155^	Renal cell carcinoma	Nivolumab Nivolumab + Ipilimumab	16:30	Before group: <20% after cutoff After group: ≥20% after cutoff	89/46	TTF, OS, ORR	mTTF: 9.5 *vs.* 4.6, *P* = 0.026 mOS: 46.3 *vs.* 41.7, *P* = 0.016 ORR: 36.0% *vs.* 29.5%, *P* = 0.157
Fernandez-Maas L *et al*^156^	Renal cell carcinoma	PD1/PD-L1 combined with/without CTLA-4	16:30	Before group: <20% after cutoff After group: ≥20% after cutoff	77/27	OS, TOT, TNT	mOS: 56.1 *vs.* 16.9, *P* = 0.01 TOT: 9.0 *vs.* 2.0, *P* = 0.01 TNT: 10.5 *vs.* 6.3, *P* = 0.06
Ramirez AMA *et al*^157^	Renal cell carcinoma	Nivolumab+Ipilimumab	16:30	Before group: <20% after cutoff After group: ≥20% after cutoff	84/43	OS, ORR	mOS: 64.8 *vs.* 46.3, *P* = 0.002 ORR: 32.8% *vs.* 22.4%, *P* = 0.04

Ortego I *et al*^158^	Metastatic urothelial cancer (mUC)	PD-1/PD-L1	16:30	Before group: <20% after cutoff After group: ≥20% after cutoff	62/26	PFS, OS, ORR	mPFS: 11.38 *vs.* 3.58, *P* = 0.001 mOS: 14.04 *vs.* 6.8, *P* = 0.001 ORR: 59.3% *vs.* 16.0%
Ruiz-Torres DA *et al*^159^	Head and neck cancer	Ipilimumab Durvalumab Pembrolizumab Nivolumab	15:00	Before group: <20% after cutoff After group: ≥20% after cutoff	49/49	OS, PFS	PFS^#^: HR = 1.57, *P* = 0.04 OS^#^: HR = 1.35, *P* = 0.26
Zheng Y *et al*^160^	Biliary tract cancer	Durvalumab Sintilimab Pembrolizumab Camrelizumab Toripalimab	16:30	Before group: <20% after cutoff After group: ≥20% after cutoff	90/49	PFS, OS, ORR	mPFS: 8.1 *vs.* 4.9, *P* = 0.006 mOS: 13.7 *vs.* 9.8, *P* = 0.01 ORR: *P* = 0.215
Naganuma A *et al*^161^	Liver Cancer	Atezolizumab+ Bevacizumab	12:00	Before group: ≥80% before cutoff After group: ≥80% after cutoff	351/400	PFS, OS, ORR	mPFS: 8.6 *vs.* 6.0, *P* = 0.006 mOS: 24.7* vs.* 21.4, *P* = 0.99 ORR: 46.2.0% *vs.* 33.2%, *P* = 0.01
Nomura M *et al*^162^	Esophageal cancer	Nivolumab	13:00	Before group: <50% after cutoff After group: ≥50% after cutoff	26/36	PFS, OS, ORR	PFS: NA, HR = 0.75, *P* = 0.303 OS: NA, HR = 0.93, *P* = 0.821 ORR: 38.5% *vs.* 25%, *P* < 0.001

CTLA-4, Cytotoxic T lymphocyte-associated protein 4; HR, hazard ratio; mPFS, Median progression-free survival; mOS, Median overall survival; NA, Not available from the reference; NSCLC, Non-small cell lung cancer; OS, Overall survival; ORR, Objective response rate; PFS, Progression-free survival; PD-1, Programmed cell death protein 1; PD-L1, Programmed cell death ligand 1; SCLC, Small cell lung cancer; ToD, Time of day; TTF, Time to failure; TOT, Time on treatment; TNT, Time to next treatment; The hash symbol (#) denotes “After versus Before.”

### Retrospective clinical studies

Among the various malignancies explored for time-dependent responses to immunotherapy, lung cancer boasts the most compelling accumulation of clinical data. The systematic and in-depth exploration of NSCLC has established a foundational framework, while nascent breakthroughs in SCLC continue to expand the clinical horizons of this burgeoning field.

In NSCLC, initial investigations into ICI infusion timing yielded inconsistent results. An early retrospective study unexpectedly found that patients receiving ICIs after 12:00 had significantly longer median PFS compared to those treated before 12:00, contrasting most subsequent findings.^140^ In contrast, research by Karaboué *et al*^141^ demonstrated that infusions administered before 12:54 significantly improved both PFS and OS, with multivariate analysis confirming early infusion as an independent protective factor. To overcome the limitations of single-center studies and draw more generalizable conclusions, Huang *et al*^142^ conducted a large-scale, dual-center retrospective study across China and France.^143^ By systematically analyzing multiple potential time cutoffs, this study was the first to definitively identify 11:30 as the most discriminative threshold. The research confirmed that administering treatment before 11:30 during the first four cycles led to significant improvements in OS and PFS, with these survival benefits remaining consistent across subgroups stratified by age, sex, and PD-L1 expression levels. This multinational collaborative study provides the most robust and actionable clinical evidence to date for chrono-immunotherapy in NSCLC. Furthermore, other studies focusing on later time, which utilized cutoffs of 16:30 and 15:00 respectively, also linked early infusion to improved PFS. Although the OS benefit in these cohorts presented only as a non-significant trend, collectively they suggest the existence of a late-afternoon “efficacy attenuation window”.^144^, ^145^, ^146^ The latest research conducted by Huang *et al*^142^ expands this paradigm to SCLC. Utilizing a 15:00 threshold, the early treatment cohort achieved significant improvements in both median PFS (mPFS) and median OS (mOS).

In contrast to the relatively consistent findings in lung cancer, the evidence in melanoma presents a more intricate and nuanced landscape. One study involving 516 patients utilized a 16:00 cutoff and associated early infusion with superior objective response rate (ORR), PFS, and OS.^147^ Similarly, a study of 299 patients using a 16:30 threshold reached comparable conclusions.^148^ However, these results appear susceptible to “survivor bias”; when the analysis was strictly confined to the first four infusions, the survival disparities vanished.^148^ Further complicating the matter, studies utilizing 11:00 or 13:00 cutoffs have produced contradictory results and suggested a potential “decoupling” between efficacy and immune-related adverse events (irAEs). Specifically, late-afternoon infusions were sometimes correlated with poorer prognosis but lower irAE incidence, or conversely, with no impact on survival but significantly more severe irAEs.^149^^,^^150^ These discrepancies underscore the unique complexity of circadian immune regulation within the melanoma microenvironment.

Evidence in gastric cancer generally aligns with the “earlier is superior” hypothesis. Two studies primarily focusing on nivolumab utilized stratification thresholds of 11:41 and 14:00, respectively, observing that early infusion significantly improved ORR, PFS, and OS.^151^^,^^152^ In contrast, another study involving diverse PD-1 inhibitor regimens and a 16:30 cutoff observed significant benefits only in OS, with no differences in PFS or ORR.^153^ This suggests that therapeutic regimen heterogeneity and the selection of temporal thresholds may significantly influence clinical outcomes in gastric cancer.

Preliminary evidence from other tumor types also follows a supportive pattern, although these studies are often constrained by limited sample sizes and research depth. In RCC, multiple studies have consistently demonstrated that morning or early-afternoon infusions significantly improve survival.^154^, ^155^, ^156^, ^157^ In metastatic urothelial carcinoma (mUC), the early-infusion group achieved a remarkable extension of more than twofold in both mPFS and mOS.^158^ Furthermore, significantly worse PFS was observed in the afternoon group for head and neck cancer,^159^ while biliary tract cancer patients in the early-infusion group saw significantly prolonged PFS and OS.^160^ In hepatocellular carcinoma and esophageal cancer, early infusions were associated with higher ORR, although significant improvements in PFS or OS have yet to be consistently established.^161^^,^^162^

Collectively, a growing body of retrospective evidence across various malignancies supports the clinical concept of chrono-immunotherapy. Lung cancer stands as the most extensively researched malignancy in this field, offering the most actionable clinical evidence and the most clearly defined optimal therapeutic windows. The identification of specific treatment thresholds—11:30 for NSCLC and 15:00 for SCLC—has established a solid foundation for prospective trials aimed at maximizing patient benefits through precision timing of administration. While consistent trends are observed across other tumor types, further validation through prospective studies utilizing standardized thresholds and larger cohorts remains essential.

### Prospective clinical studies

Zhang *et al*^100^ conducted a prospective, randomized, single-center, open-label phase III trial that enrolled 210 treatment-naïve patients with stage IIIC–IV non-small-cell lung cancer lacking sensitizing *EGFR/ALK/ROS1* mutations. Patients were randomized 1:1 to an “early” or “late” infusion group; group assignment was based on a retrospective analysis of institutional data that identified the optimal time cutoff: administration of ICI prior to 15:00 (early) versus at/after 15:00 (late) during the first four treatment cycles. The primary endpoint was PFS assessed by a blinded independent review committee; secondary endpoints included OS and ORR. Results showed mPFS of 11.3 months versus 5.7 months (early *vs.* late; HR = 0.40, *P* < 0.001), median OS of 28.0 months versus 16.8 months (early *vs.* late; HR = 0.42, *P* < 0.001), and ORR of 69.5% versus 56.2% (*P* = 0.046). This study advances chronotherapy in oncology from retrospective observation to prospective validation and provides an evidence-based foundation for individualized, precision-timed treatment strategies.

With the continued accumulation of retrospective clinical evidence, chronomodulated immunotherapy in oncology has entered a pivotal phase of large-scale prospective validation. As summarized in Table 2, multiple ongoing and planned registry-based clinical trials highlight the latest advances in this field, spanning diverse tumor types and treatment settings. In advanced solid tumors, current efforts focus on dual-immune checkpoint blockade (e.g., NCT07155317 in melanoma and NCT07338981 in advanced renal cancer) as well as innovative combinations such as ICIs plus antibody–drug conjugates (ADCs) (e.g., NCT07346053 in advanced bladder cancer). Notably, investigation of chronotherapy has moved earlier in the disease course into the perioperative neoadjuvant setting, including studies in triple-negative breast cancer (NCT06880029) and NSCLC (NCT07251582), both using pathological complete response (pCR) as a key efficacy endpoint. In addition, the pan-tumor advanced solid tumor trial (NCT07224971) is designed to further test the generalizability of the timing effect. By implementing predefined dosing time cutoffs (e.g., 11:00, 11:30, or 12:00) and incorporating survival endpoints such as PFS and OS, these studies are expected to generate definitive evidence to inform clinical practice, facilitating the translation of chronomodulated immunotherapy from concept to implementation and advancing toward precision, individualized care.

**Table 2:** Prospective registry-based clinical study.

Study type (NCT number)	Study phase	Study design	Condition	Intervention	Study arms	Enrollment (n)	Primary endpoint	Start and completion date	Recruitment status
Clinical trial (NCT07155317)	Phase 2	Prospective randomized study	Stage IV or unresectable melanoma	Nivolumab + Ipilimumab	Group A: 08:00–11:00 Group B: 11:00–14:00 Group C: 14:00–17:00	99	PFS, OS, ORR, AE	2025.11.29-–2027.11.31	Recruiting
Clinical trial (NCT06880029)	Phase 1/Phase 2	Prospective randomized study	Triple negative breast cancer	Neoadjuvant pembrolizumab	AM: before noon PM: after noon	20	pCR Rate	2024.9.1–2025.9.1	Recruiting
Clinical trial (NCT07224971)	Phase 2	Prospective randomized study	Various advanced/metastatic malignancies (focusing on solid tumors such as NSCLC)	Anti-PD-1/PD-L1 immunotherapy (including pembrolizumab, nivolumab)	AM: before 11:00 PM: after 12:00	350	PFS	2025.12.2–2030.5.1	Recruiting
Clinical trial (NCT07338981)	Phase 3	Randomized controlled study	Advanced kidney cancer	Nivolumab + Ipilimumab	AM: before 11:30 PM: after 13:30	142	PFS, OS, ORR, TTF	2026.4–2032.12	Not yet recruiting
Clinical trial (NCT07346053)	Phase 3	Randomized controlled study	Advanced bladder cancer	Enfortumab Vedotin + Pembrolizumab	AM: before 11:30 PM: after 13:30	224	ORR, PFS, OS, TTF	2026.5–2032.12	Not yet recruiting
Clinical trial (NCT07251582)	Phase 3	Prospective randomized study	Resectable stage II-III NSCLC	Toripalimab or pembrolizumab + platinum-based chemotherapy	AM: 08:00–11:00 PM: 15:00–18:00	156	pCR	2025.11.7–2029.5.31	Recruiting

AE, Adverse event; NSCLC, Non-small cell lung cancer; OS, Overall survival; ORR, Objective response rate; PFS, Progression-free survival; pCR, Pathological complete response; PD-1, Programmed cell death protein 1; PD-L1, Programmed cell death ligand 1; TTF, Time to failure.

### Circadian rhythms and immunotherapy-related adverse events in lung cancer

Emerging evidence suggests that the tumor itself can induce profound endocrine and metabolic dysregulation within the host.^110^ This systemic disruption may extend to the circadian timing system, which governs numerous physiological processes. Consequently, circadian rhythms not only significantly influence the efficacy of immunotherapy but may also affect the incidence and severity of irAEs. However, direct clinical evidence regarding whether adjusting the infusion timing of ICIs can reliably alter their toxicity profile remains limited and inconsistent. In studies reporting adverse events, some suggested that earlier administration was associated with a higher overall incidence of toxicities.

Existing clinical data on the impact of infusion timing on toxicity broadly show two relatively consistent patterns. One group of studies suggests that earlier administration improves efficacy but is accompanied by increased toxicity, indicating a parallel relationship between efficacy and toxicity. Catozzi *et al*^163^ reported in a mixed-cancer cohort that patients receiving earlier treatment had better OS and ORR, but also experienced higher-grade toxicities. Similarly, in advanced melanoma, earlier infusion was associated with longer OS and PFS but a higher incidence of irAEs. Karaboue *et al*^141^ demonstrated in NSCLC that earlier administration improved OS and PFS, but was accompanied by increased dermatologic toxicity. Yeung *et al*^150^ found that in stage IV melanoma, grade 3–4 toxicities were reported exclusively in the early-treatment group, which nonetheless had longer OS. Another group of studies found that although earlier administration improved survival outcomes, toxicity profiles did not differ significantly between groups, suggesting that in certain populations and regimens, optimizing infusion timing may enhance efficacy without substantially increasing adverse events.^124^^,^^143^^,^^152^^,^^159^ Furthermore, individual studies have shown that the temporal distribution of different toxicity types is not entirely consistent. For instance, in one NSCLC study, the most common adverse events were fatigue and dermatologic toxicity; the frequency of grade 3–4 fatigue was higher in the late-treatment group (14.9% *vs.* 6.3%), while grade 2–3 dermatologic toxicity occurred more frequently in the early-treatment group (31.9% *vs.* 12.7%).^141^ Overall, evidence regarding the relationship between ICI infusion timing and toxicity remains inconsistent, potentially influenced by factors such as tumor type, patient baseline characteristics, treatment regimens, and toxicity assessment methods, underscoring the need for more prospective, randomized studies to clarify this issue.

Several critical challenges remain in this field. First, the lack of standardized cut-off times (ranging from 11:30 to 16:30) and the reliance on retrospective rather than prospective, biology-driven hypotheses make replication and integration of results difficult. Second, most studies are retrospective and therefore subject to confounding; for instance, patients treated in the morning may have better baseline health status or access to more comprehensive care. Third, infusion time has not been uniformly defined. Some studies classify groups based on the proportion of doses given before or after a cutoff, whereas others focus only on the first few cycles, further complicating comparisons. Most importantly, there is still a lack of large-scale prospective randomized controlled trials to establish a causal relationship between infusion timing and treatment efficacy or toxicity. The underlying biological mechanisms of circadian influence also remain to be fully elucidated. Future studies should therefore prioritize prospective clinical trials with systematic recording of infusion timing and time-dependent toxicity data, in order to define optimal infusion strategies across tumor types and treatment regimens, and to provide high-level evidence for clinical practice.

## Future research directions: a focus on chronotherapy in lung cancer

Chronotherapy, which integrates circadian rhythms with cancer immunotherapy, is rapidly emerging as a novel strategy to enhance antitumor efficacy while reducing treatment-related toxicity. In lung cancer, the available retrospective evidence is most robust and the exploration of optimal treatment windows is most advanced, providing a solid foundation for prospective investigations. To translate this field from descriptive observation to clinical application, future research should prioritize carefully designed prospective trials focused on different lung cancer stages, methodological standardization, mechanistic studies, and translational clinical implementation.

### Conducting well-designed prospective clinical trials with a focus on lung cancer

The highest priority is to conduct large-scale, multicenter prospective randomized controlled trials (RCTs) to generate level I evidence on the impact of infusion timing in immunotherapy.^164^ These trials should establish predefined time cut-offs based on circadian biology rather than retrospective *post hoc* divisions. Adequate statistical power is needed to detect differences in primary endpoints (OS, PFS) and secondary endpoints (ORR, quality of life) across treatment groups.

Building on this priority, future research directions can be further expanded in the following areas: First, it is necessary to determine whether controlling the timing of the first few infusions is essential for optimizing patient survival as retrospective data suggest that infusion timing during the initial treatment cycles may critically influence long-term survival outcomes, with multiple studies in NSCLC demonstrating that morning infusions are associated with superior survival (e.g., 11:30 and 11:00 as discriminative thresholds for the first four cycles and first infusion, respectively).^165^ Second, in NSCLC, the concept of chronotherapy can be advanced to the neoadjuvant setting, exploring the optimal timing of perioperative immunotherapy with pCR as the primary endpoint, thereby optimizing perioperative treatment strategies for lung cancer. Third, in SCLC, based on positive retrospective evidence, prospective phase III randomized controlled trials are urgently needed to validate the clinical value of chronomodulated immunotherapy in extensive-stage SCLC. Furthermore, trials should be extended to other innovative therapies in lung cancer, including novel combination regimens (e.g., ICIs combined with anti-angiogenic agents or chemotherapy) and bispecific antibodies, to determine the broad applicability of circadian optimization strategies in comprehensive lung cancer treatment.

### Establishing a standardized data collection and consensus definition system using lung cancer as a paradigm

A major challenge in synthesizing evidence across studies is the substantial heterogeneity in data collection and definition standards, an issue particularly pronounced in chronotherapy research for lung cancer. To build a reliable evidence base for chronotherapy, standardization must be achieved at the following three levels.

**Standardization of temporal definitions**. It is recommended that “early” and “late” treatment windows be clearly defined based on physiological rhythm characteristics rather than convenience. In lung cancer research, cutoffs validated by large-scale retrospective studies (e.g., 11:30 for NSCLC and 15:00 for SCLC) should be adopted as reference standards for temporal definitions. Given inter-individual variability, multiple time-partitioning methods should be explored within the same study through sensitivity analyses, and individual chronotype should be incorporated as a stratification or adjustment factor to more accurately evaluate the impact of infusion timing on clinical outcomes. The ultimate goal is to establish an optimal temporal definition standard that balances generalizability and individualization by comparing the predictive performance of different time-partitioning approaches, thereby ensuring comparability across studies.

**Standardization of data acquisition**. Electronic medical record systems must accurately document the actual start and end time of each drug administration while systematically collecting patient circadian rhythm parameters. In lung cancer clinical practice and research, actigraphy is recommended for monitoring sleep–wake patterns, and standardized questionnaires (e.g., the Munich Chrono Type Questionnaire) should be used to assess individual chronotype.^166^ Particular attention should be paid to the fact that patients with advanced lung cancer often experience sleep fragmentation and circadian disruption due to symptoms such as dyspnea, cancer-related fatigue, and cough; these clinical features should be considered essential components of circadian parameter collection and analysis.

**Standardization of adverse event recording**. IrAEs should be documented using a time-anchored model that includes toxicity type, time of onset, and its correlation with treatment cycles. Given the relatively high incidence of potentially life-threatening immune-related pneumonitis in lung cancer patients, the temporal relationship between onset time, severity, and infusion timing should be systematically recorded to provide data support for investigating the impact of temporal factors on toxicity profiles.

This standardized system will lay the foundation for establishing a high-quality time-series treatment database for lung cancer, facilitating the translation from population-level patterns to individualized chronotherapy strategies. The standards established using lung cancer as a paradigm can be extended to other tumor types in the future, forming a unified research framework for chrono-immunotherapy.

### Developing a multidimensional circadian intervention synergy program

Based on molecular mechanisms of circadian rhythm regulation, we propose the concept of “Active Rhythm Optimization,” extending traditional chronotherapy into a multimodal circadian intervention system. This system comprises three tiers.

Basic synchronization tier: Prior to initiating time-sensitive treatments, standardized rhythm pre-conditioning should be implemented for patients, including morning fixed light exposure, a time-restricted feeding window (NCT06296823), and low-dose melatonin supplementation adjusted according to individual sleep phase. For lung cancer patients, given the high prevalence of cachexia and anorexia, time-restricted feeding protocols should be individualized under the guidance of nutritionists to avoid exacerbating malnutrition. Therapeutic synergy tier: Building upon core treatments such as ICIs, precisely aligning drug infusion timing with the patient’s peak immune function rhythm and enhance rhythm amplitude using small-molecule agonists targeting key clock genes may further improve treatment outcomes. Preclinical studies have demonstrated that small-molecule compounds targeting REV-ERB or ROR in lung cancer models significantly influence tumor growth and immune cell infiltration, providing a theoretical foundation for combination strategies in lung cancer chronotherapy. Dynamic adjustment tier: A wearable device-driven real-time monitoring system should be developed to dynamically fine-tune the treatment time window by continuously tracking physiological rhythm markers such as heart rate variability and skin temperature.^167^ In lung cancer patients, pulse oximetry monitoring may be further integrated to assess the impact of respiratory function on sleep rhythm in real time, facilitating dynamic optimization of dosing schedules.

### Constructing a translational pathway from evidence to clinical practice

Building upon an increasingly robust evidentiary base and standardized data acquisition systems, the critical next step is to transition from population-averaged scheduling to truly individualized precision chronotherapy. We propose lung cancer as a strategic entry point to systematically establish and validate an intelligent Clinical Decision Support System (CDSS) that integrates multi-dimensional circadian data.

First, the establishment of individualized circadian phenotyping and predictive models for lung cancer patients is essential. This requires the integration of sleep–wake rhythms captured by wearable devices, patient-reported chronotypes and symptomatic data, and the circadian fluctuation patterns of key immune and endocrine biomarkers. Additionally, the rhythmic characteristics of lung cancer-specific markers should be incorporated. Utilizing machine learning-driven data fusion techniques, these inputs will generate quantified “individualized immune-metabolic circadian phase maps” to precisely predict the dynamic daily windows for optimal immune activity and drug metabolism for each patient.

Second, a lung cancer-oriented CDSS platform must be developed and prospectively validated. This platform should be deeply integrated with Hospital Information Systems (HIS) to automatically process multi-source data and provide clinicians with personalized therapeutic window recommendations, supplemented by confidence levels. Its clinical superiority must be rigorously evaluated through RCTs comparing system-guided individualized timing against the standard “uniform morning administration” strategy.

Third, dynamic and adaptive temporal adjustment strategies for lung cancer management should be explored. Given that therapeutic interventions and disease progression can dynamically reshape a patient’s biological rhythms, the system must possess continuous learning and iterative capabilities. Future research should focus on strategies that reassess the patient’s circadian status at preset treatment intervals and adjust subsequent dosing schedules accordingly. This will drive the evolution of chronotherapy from static protocols toward a longitudinal “adaptive precision chronotherapy” management paradigm.

## Conclusion

This article systematically delineates the multi-layered, dynamic interplay between circadian rhythms and tumor immunity, positioning the biological clock as a central regulator of tumor initiation and progression, immune surveillance, and therapeutic responsiveness.

At the molecular level, core clock genes maintain physiological rhythms and organismal homeostasis through interconnected transcription–translation feedback loops. Simultaneously, these genes directly influence tumor cell behavior, regulating key processes such as the cell cycle, apoptosis, and autophagy. Dysregulation of these clock components acts as a critical intrinsic driver of malignant progression. Within the tumor immune microenvironment, circadian rhythms program immune function in a spatiotemporally defined manner. They shape the activation and infiltration kinetics of T cells, the cyclical cytotoxicity of NK cells, the metabolic and migratory rhythms of antigen-presenting cells, and the balance of macrophage polarization. This rhythmic organization of the immune system, in turn, critically determines the magnitude and quality of anti-tumor immunity.

At the translational and clinical level, accumulating evidence indicates that the efficacy of ICIs is significantly time-of-day dependent. Retrospective and prospective studies suggest that, in specific malignancies (e.g., NSCLC and gastric cancer), morning or pre-noon administration is associated with improved survival outcomes. These findings provide an evidence-based rationale for chrono-immunotherapy and underscore that “when to treat” can be as consequential as “what to treat.”

To move chronotherapy from concept to routine care, future efforts should establish an integrated development pathway spanning mechanistic investigation, clinical validation, and implementation. A priority is the conduct of rhythm-informed prospective trials to generate high-level evidence, alongside standardized frameworks for temporal data capture and assessment. Beyond simply adjusting dosing times, the field should advance toward a multidimensional “active rhythm optimization” strategy integrating light-based interventions, chronobiotic agents, and real-time biomarker monitoring. Ultimately, the development of intelligent clinical decision-support tools, evidence-based guidelines, and regulatory recognition will be essential to enable standardized yet individualized chronotherapy.

In summary, incorporating chronobiological principles into tumor immunotherapy not only deepens our understanding of tumor–immune interactions but also introduces a new strategic dimension for enhancing efficacy and reducing toxicity through optimized treatment timing. This evolution signals a shift from “static” to “dynamic” precision oncology and offers a promising opportunity to improve patient outcomes.

## Funding

This work was supported by the National Natural Science Foundation of China (No. 82573423). The funding agencies had no role in the study design, data collection, analysis, interpretation, manuscript writing, and the decision to submit the article for publication.

## CRediT authorship contribution statement

**Fang Tian:** Writing – review & editing, Writing – original draft, Investigation. **Songkai Wang:** Writing – original draft, Visualization, Investigation. **Zhe Huang:** Writing – review & editing, Methodology, Conceptualization. **Zhaohui Ruan:** Writing – review & editing, Methodology, Conceptualization. **Jiacheng Dai:** Supervision, Methodology. **Huan Yan:** Supervision, Methodology. **Jiao Huang:** Data curation. **Dan Yang:** Data curation. **Xiaomei Li:** Supervision, Methodology, Conceptualization. **Liang Zeng:** Supervision. **Qinqin Xu:** Writing – review & editing, Supervision. **Yongchang Zhang:** Visualization, Methodology, Conceptualization.

## Declaration of competing interest

The authors declare that they have no known competing financial interests or personal relationships that could have appeared to influence the work reported in this paper.
